# *Lactobacillus plantarum* L168 improves hyperoxia-induced pulmonary inflammation and hypoalveolarization in a rat model of bronchopulmonary dysplasia

**DOI:** 10.1038/s41522-024-00504-w

**Published:** 2024-03-29

**Authors:** Xian Shen, Zhaocong Yang, Qiang Wang, Xu Chen, Qihui Zhu, Zhi Liu, Nishant Patel, Xingyin Liu, Xuming Mo

**Affiliations:** 1 Department of Neonatology, Children’s Hospital of Nanjing Medical University, Nanjing, China; 2 Department of Cardiothoracic Surgery, Children’s Hospital of Nanjing Medical University, Nanjing, China; 3 State Key Laboratory of Reproductive Medicine, Key Laboratory of Pathogen of Jiangsu Province, Key Laboratory of Human Functional Genomics of Jiangsu Province Center of Global Health, Nanjing Medical University, Nanjing, China

**Keywords:** Applied microbiology, Clinical microbiology

## Abstract

Alteration of gut microbiota can affect chronic lung diseases, such as asthma and chronic obstructive pulmonary disease, through abnormal immune and inflammatory responses. Previous studies have shown a feasible connection between gut microbiota and bronchopulmonary dysplasia (BPD) in preterm infants. However, whether BPD can be ameliorated by restoring the gut microbiota remains unclear. In preterm infants with BPD, we found variance in the diversity and structure of gut microbiota. Similarly, BPD rats showed gut dysbiosis, characterized by a deficiency of *Lactobacillus*, which was abundant in normal rats. We therefore explored the effect and potential mechanism of action of a probiotic strain, *Lactobacillus plantarum* L168, in improving BPD. The BPD rats were treated with *L. plantarum* L168 by gavage for 2 weeks, and the effect was evaluated by lung histopathology, lung function, and serum inflammatory markers. Subsequently, we observed reduced lung injury and improved lung development in BPD rats exposed to *L. plantarum* L168. Further evaluation revealed that *L. plantarum* L168 improved intestinal permeability in BPD rats. Serum metabolomics showed altered inflammation-associated metabolites following *L. plantarum* L168 intervention, notably a marked increase in anti-inflammatory metabolites. In agreement with the metabolites analysis, RNA-seq analysis of the intestine and lung showed that inflammation and immune-related genes were down-regulated. Based on the information from RNA-seq, we validated that *L. plantarum* L168 might improve BPD relating to down-regulation of TLR4 /NF-κB /CCL4 pathway. Together, our findings suggest the potential of *L. plantarum* L168 to provide probiotic-based therapeutic strategies for BPD.

## Introduction

BPD, also called chronic lung disease, is a syndrome of chronic lung injury resulting in impaired microvascular development and alveolarization of the lungs in premature babies. In premature infants, BPD is a leading cause of morbidity and mortality^1–3^. BPD is not simply a consequence of lung immaturity, but also the result of aberrant regulation and abnormal repair of lung inflammation^1–3^, which leads babies to require ventilation support and/or supplemental oxygen for months to years. With the increase in the survival rate of extremely low gestational age newborns, the incidence of BPD is also escalating^4,5^. Globally, BPD is estimated to occur in 11–50% of premature infants with gestational age less than 30 weeks^3^, even 80.7% for birth weight less than 750 g^6^. BPD not only leads to oxygen dependence, pulmonary hypertension, and prolonged hospitalization in the short term, but also increases the risk of asthma and chronic obstructive pulmonary disease (COPD) in the long term^7–9^. Currently, the treatment of BPD mainly involves comprehensive therapies such as nutritional support, respiratory management, and anti-inflammatory treatment^2^. The lack of target drug interventions is the greatest challenge in clinical practice.

Meanwhile, increasing evidence has shown that the gut microbiota continuously interacted with many other important organs of the host, such as the brain and bone marrow^10–12^. The imbalance of gut microbiota can affect distant target organs through multiple pathways, including damaging the intestinal mucosal barrier and affecting the metabolites of gut microbiota, host metabolome, pro-inflammatory immune response, and energy metabolism^13^. The gut-lung axis has been a hotspot in the study of chronic lung diseases in recent years, such as asthma and COPD^14–16^. However, only a few clinical and basic studies have been conducted regarding the relationship between gut microbiota and BPD^17,18^. The effect of intervening with gut microbiota on BPD still needs further exploration.

Probiotics can promote the growth of symbiotic bacteria and keep the balance of the intestinal microecological system. *Lactobacillus* is a probiotic belonging to the Firmicutes phylum, which can protect the intestinal mucosal barrier, reduce inflammation, and promote weight gain^19,20^. A few *Lactobacillus* strains have been reported to stimulate regulatory T cell development and reduce inflammatory cytokine secretion^21^. Li et al. found that *Lactobacillus reuteri* (*L. reuteri*) could improve the immune environment of the lungs by modulating the gut microbiota, thereby decreasing the asthma risk^22^. Whereas Griet et al. found that LPS-induced proinflammatory cytokine secretion, inflammatory cell recruitment to the airway, and inflammation-related lung tissue damage were significantly decreased in mice treated with *L. reuteri* CRL1098 soluble factor^23^. *L. plantarum* L168, isolated by Liu’s Lab^24^, has been shown to have several beneficial properties, including restoring gut microbiota, modulating the concentration of the neurotransmitter serotonin, and improving impaired behavior in flies^24^. Nevertheless, the role of *Lactobacillus* in BPD has not been reported.

In this study, we first explored the gut microbiota characteristics of BPD premature infants and hyperoxia-induced BPD rats. Next, we investigated the ameliorating effect of *L. plantarum* L168 on hyperoxia-induced BPD rats. We further explored the feasibility of probiotics or modulating pathways as therapeutic targets for BPD.

## Results

### BPD preterm infants show alteration of gut microbiota compared with non-BPD preterm infants

**Fig. 1:**
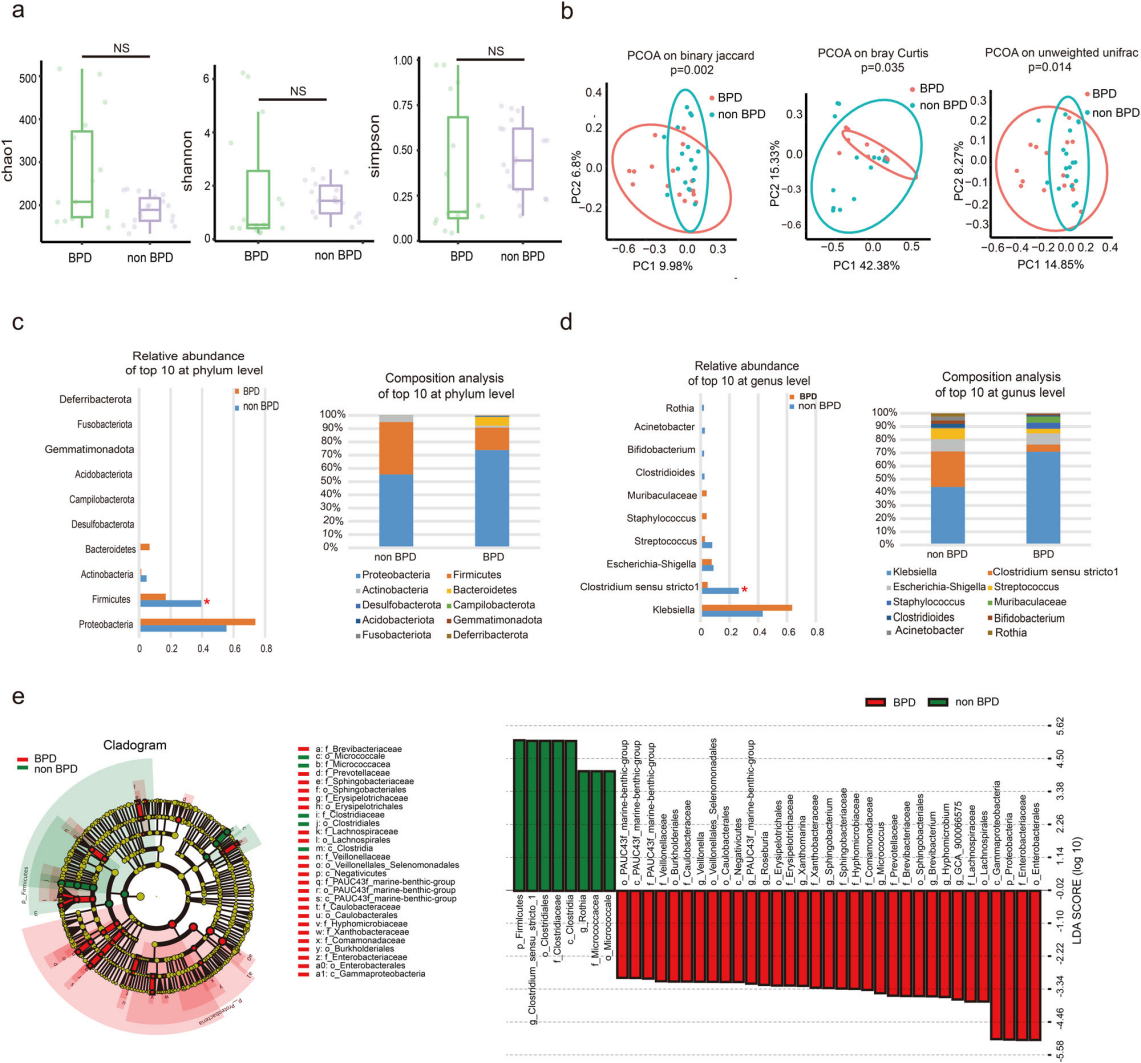
BPD preterm infants show alteration of gut microbiota compared with non-BPD preterm infants. Microbiome composition was determined by 16S rRNA gene sequencing. **a** Chao, Shannon and Simpson of α-diversity index in BPD (*n* = 15) and non-BPD (*n* = 18) groups. **b** Principal Co-ordinates Analysis (PCoA) of β-diversity index in BPD and non-BPD groups. **c** Comparison of relative abundance and composition of top 10 at phylum level. **d** Comparison of relative abundance and composition of top 10 at genus level. **e** Cladogram for differentially abundant taxa and linear discriminant analysis Effect Size (LEfSe) for differential taxa between BPD and non-BPD groups (*p* < 0.05, LDA score (log10) > 3.0). Data are expressed as the means ± SD (**a**) and unpaired *t*-test was performed. *p < 0.05; and NS not statistically significant.

There were no significant differences between BPD and non-BPD preterm infants with regards to birth weight, birth mode, premature rupture of membranes, and antenatal use of antibiotics (Table 1). Although almost all preterm infants in the two groups were given antibiotics after birth because of their serious disease, no significant differences were found in the utilization rate of antibiotics between the two groups. Other factors, including breastfeeding and necrotizing enterocolitis, did not differ significantly between the two groups. The durations of mechanical ventilation, non-invasive ventilation and oxygen therapy in the BPD group were significant longer than that in the non-BPD group, respectively. The differences in requirements of oxygen and ventilation were mainly related to the disease severity of premature infants after birth, and were also important pathogenic factors for the development of BPD. 16S rRNA gene sequencing analysis showed there was no significant difference in α-diversity between BPD and non-BPD groups (Fig. 1a), while β-diversity displayed significant differences between groups (Fig. 1b). Proteobacteria, Firmicutes, Actinobacteria and Bacteroidetes were the main phylums in the premature babies, and the relative abundances were different between the two groups. Notably, the relative abundance of Firmicutes is significantly higher in the non-BPD group (Fig. 1c). At the genus level, *Clostridium sensu stricto 1* was significantly lower in the BPD group (Fig. 1d). Linear Discriminant analysis Effect Size (LEfSe) showed an obvious alteration of microbiota characterized by enrichment advantages of *Clostridium* and *Rothia* in the non-BPD group. However, *Veillonella*, *Roseburia*, *Micrococcus*, *Xanthomarina* were significantly enriched in the BPD group (Fig. 1e). These results indicated that BPD infants showed an alteration of gut microbiota composition compared with non-BPD infants, suggesting that there might be a relationship between gut microbiota and BPD through the gut-lung axis.

**Table 1:** Clinical characteristics of the prematures by BPD status

	Non-BPD group (*n* = 18)	BPD group (*n* = 15)	${\boldsymbol{\chi}}^2$/*t*/*Z*	*p* value
Gestational age (weeks)	30.17 ± 1.25	28.93 ± 1.24	2.843	0.008
Birth weight (grams)	1415.00 ± 283.57	1244.00 ± 231.76	1.871	0.071
Gender (male, %)	10 (55.6)	13 (86.7)	3.750	0.053
Cesarean delivery (%)	10 (55.6)	10 (66.7)	0.423	0.515
Antenatal corticosteroid (%)	6 (33.3)	4 (26.7)	0.524	0.769
PROM > 18 h (%)	6 (33.3)	5 (33.3)	0.000	1.000
5 min Apgar Score ≤ 7 (%)	0 (0.0)	3 (20.0)		0.083
Antenatal use of antibiotics (%)	3 (16.7)	4 (26.7)		0.674
NEC (≥IB, %)	2 (11.1)	5 (33.3)		0.203
Postnatal use of antibiotics within 28 days after birth (%)	17 (94.4)	15 (100.0)	0.859	0.354
Postnatal use of antibiotics within a week before stool collection (%)	4 (22.2)	7 (46.7)		0.163
Breastfeeding (%)	9 (50.0)	5 (33.3)	0.930	0.335
Weight at 28 days after birth (grams)	1897.83 ± 346.55	1676.00 ± 269.52	2.067	0.047
Duration of total enteral nutrition during hospitalization (days)	23 (15.5, 31.0)	36 (26.0, 41.0)	−2.226	0.026
Duration of invasive ventilation (h)	0 (0,31.75)	135 (0,252.0)	−3.190	0.001
Duration of non-invasive ventilation (h)	124.5 (68.5,253.5)	390 (154.0,870.0)	−2.603	0.009
Duration of oxygen therapy (h)	277.5 (234.0,822.5)	1023 (834.0,1298.0)	−4.049	<0.001

### BPD rats show gut dysbiosis compared with control rats

**Fig. 2:**
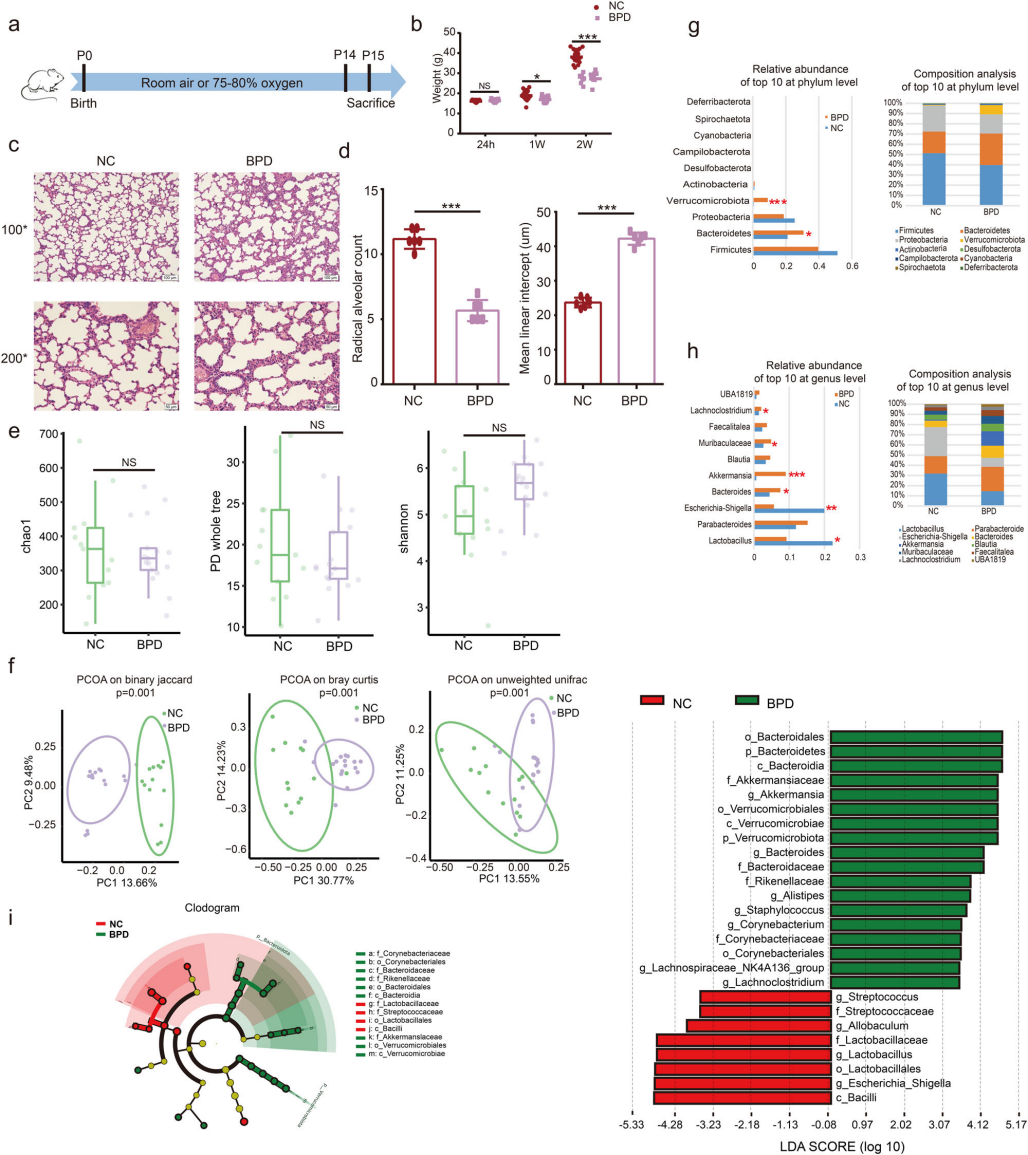
BPD rats show gut dysbiosis compared with control rats. **a** Experimental strategy. **b** Quantitative analysis of body weight in normal control and BPD rats (*n* = 12–15 each group). **c** Representative photomicrographs of HE-stained lung sections (scale bar, 100 μm and 50 μm) (*n* = 6 each group). **d** Comparison of radial alveolar counts (RAC) and mean linear intercept (MLI) between normal control and BPD rats (*n* = 6 each group). **e** Chao, PD whole tree and Shannon of α-diversity index in normal control (*n* = 13) and BPD rats (*n* = 17). **f** PCoA of β-diversity index in normal control and BPD rats. **g** Comparison of relative abundance and composition of top 10 at phylum level. **h** Comparison of relative abundance and composition of top 10 at genus level. **i** Cladogram for differentially abundant taxa and LEfSe analysis for differential taxa between normal control and BPD rats. (*p* < 0.05, LDA score (log10) > 3.0). Data are expressed as the means ± SD (**b**, **d**, **e**) and unpaired *t*-test was performed. ***p < 0.001; **p < 0.01; *p < 0.05; and NS not statistically significant.

Based on the above results, we next sought to explore the characteristics of gut microbiota in BPD rats. We used a well-established rat model of neonatal BPD induced by hyperoxia^25^. The newborn rats with their mothers were randomly grouped within 24 h after birth, and exposed to either hyperoxia (FiO_2_ 75–80%) or normoxia (FiO_2_ 21%) for 2 weeks (Fig. 2a). Compared with the normoxia-exposed rats, the body weights of the hyperoxia-exposed rats were significantly lower (Fig. 2b). HE staining of the lungs showed impaired alveolarization, large airspaces and thickened alveolar septation in the hyperoxia-exposed group (Fig. 2c). Similarly, the radial alveolar counts (RAC), which is an important indicator for the evaluation of lung development, was significantly lower, and the mean linear intercept (MLI), which represents the average internal diameter of the alveolar space, was significantly higher in the hyperoxia-exposed group (Fig. 2d). All of these were consistent with the lung histological characteristics of BPD and verified the successful construction of the BPD model.

After verifying the BPD model, we then analyzed the characteristics of the gut microbiota. There was no significant difference in α-diversity between the two groups (Fig. 2e), but the β-diversity showed significant differences (Fig. 2f). Firmicutes, Bacteroidetes, Proteobacteria and Verrucomicrobiota were the main phylums in all rats, whereas the relative abundances were different between the two groups. The relative abundances of Bacteroidetes and Verrucomicrobiota were significantly higher in the BPD rats (Fig. 2g). At the genus level, *Lactobacillus* and *Escherichia-Shigella* were obviously lower in BPD rats, while *Bacteroides*, *Akkermansia*, *Muribaculaceae* and *Lachnoclostridium* were significant higher in BPD rats (Fig. 2h). Further LEfSe analysis also showed the enrichment of *Lactobacillus* in the normal control rats (Fig. 2i). These results implicated that BPD rats had altered gut microbiota similar to BPD infants.

### Oral administration of *Lactobacillus plantarum* L168 attenuates BPD

**Fig. 3:**
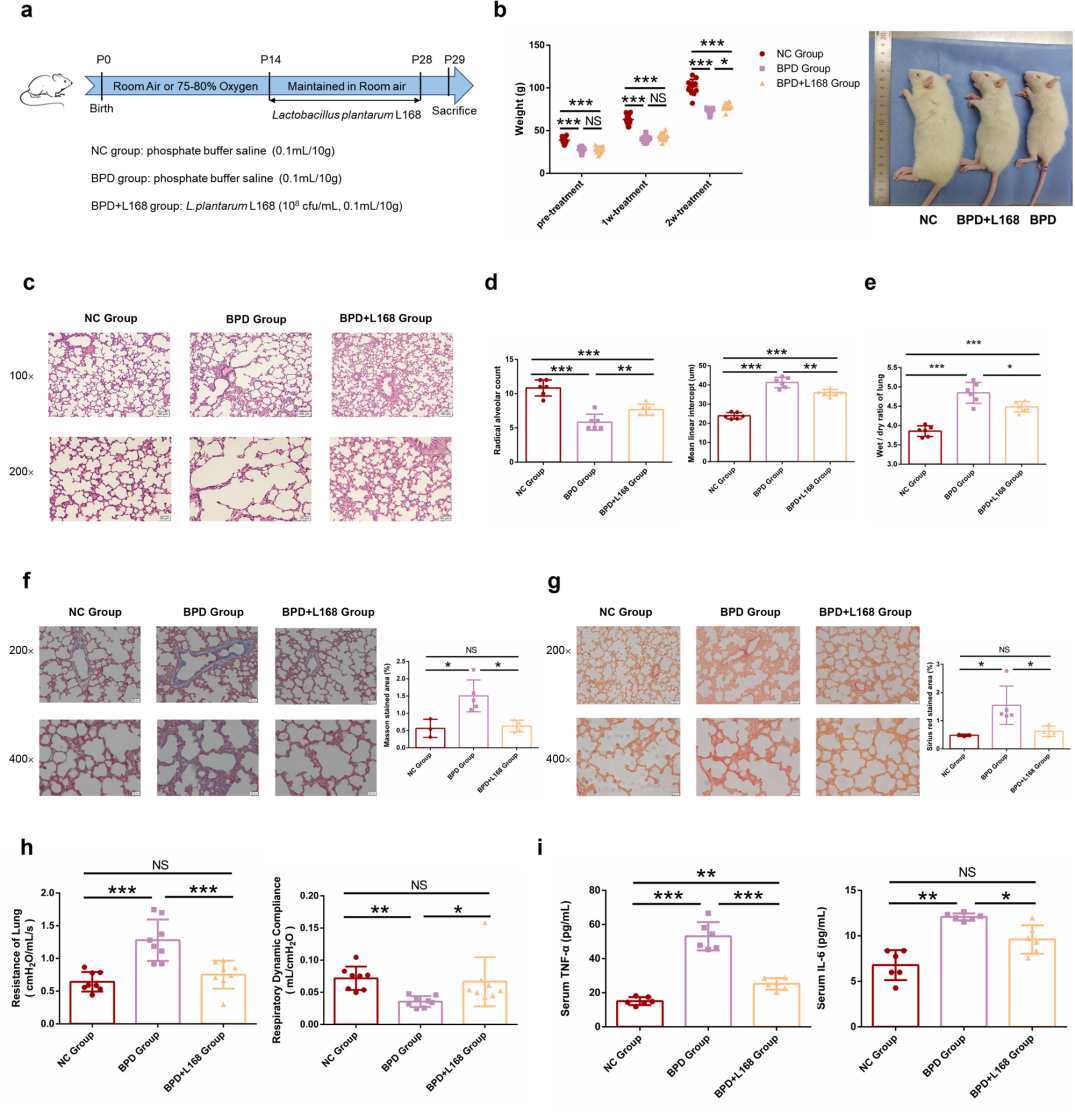
Oral *Lactobacillus plantarum* L168 attenuates BPD. **a** Experimental strategy. **b** Quantitative analysis of body weight in normal control, BPD and BPD + L168 rats (*n* = 10–12 each group). **c** Representative photomicrographs of HE-stained lung sections (scale bar, 100 μm and 50 μm) (*n* = 6 each group). **d** Comparison of RAC and MLI in normal control, BPD and BPD + L168 rats (*n* = 6 each group). **e** Comparison of wet/dry ratio of lung in normal control, BPD and BPD + L168 rats (*n* = 6 each group). **f** Representative photomicrographs of Masson-stained lung sections (scale bar, 50 μm and 20 μm) (*n* = 3–5 each group). **g** Representative photomicrographs of Sirius red-stained lung sections (scale bar, 50 μm and 20 μm) (*n* = 3–5 each group). **h** Comparison of pulmonary functions in normal control, BPD and BPD + L168 rats: airway resistance (R) and pulmonary compliance (C) (*n* = 8 each group). **i** The levels of serum TNF-α and IL-6 in normal control, BPD and BPD + L168 rats (*n* = 6 each group). Data are expressed as the means ± SD (**b**) and unpaired *t*-test was performed. Data are expressed as the means ± SD (**d**–**i**) and one-way ANOVA was performed. ***p < 0.001; **p < 0.01; *p < 0.05; and NS not statistically significant.

The 16S rRNA gene sequencing analysis showed that *Lactobacillus* was enriched in normal control rats but significantly lower in BPD rats. Therefore, we treated the BPD rats with our previous identified strain, *L. plantarum* L168^24^, and then explored the effects of *L. plantarum* L168 on BPD. We orally gavaged male BPD rats with *L. plantarum* L168 (10^8^ cfu/ml, 0.1 ml/10 g) or PBS (0.1 ml/10 g) once a day for 2 weeks (Fig. 3a). After *L. plantarum* L168 treatment for 2 weeks, the body weights were higher than the BPD untreated rats, still lower than the normal rats (Fig. 3b). We further evaluated the improvement of BPD by lung histopathology. Compared with the BPD untreated group, the number of alveoli was greater, the enlargement of alveolar cavity was improved, and the thickening of alveolar septum was reduced with *L. plantarum* L168 (Fig. 3c). After treatment of *L. plantarum* L168, the BPD rats had less lung injury with increased RAC and decreased MLI (Fig. 3d). Compared with the BPD untreated group, the ratio of wet/dry weight of lung tissue was significantly reduced with *L. plantarum* L168 (Fig. 3e), suggesting that *L. plantarum* L168 can alleviate pulmonary edema. Masson and Sirius red staining showed collagen fibrosis deposition was much less in the lung after *L. plantarum* L168 treatment for 2 weeks (Fig. 3f, g). Following, we assessed the pulmonary function of rats. Compared with the normal control rats, the airway resistance in BPD group was clearly elevated, whereas, the elevation was suppressed by *L. plantarum* L168 treatment. In addition, the pulmonary compliance of BPD rats was significantly lower than that of normal control rats, whereas it was significantly improved by *L. plantarum* L168 treatment (Fig. 3h). *L. plantarum* L168 treatment also resulted in lower serum TNF-α and IL-6 (Fig. 3i), indicating that *L. plantarum* L168 treatment can reduce the inflammatory response. Therefore, *L. plantarum* L168 played a protective role in BPD.

### Oral administration of *Lactobacillus plantarum* L168 improves the barrier function of gut in BPD rats

**Fig. 4:**
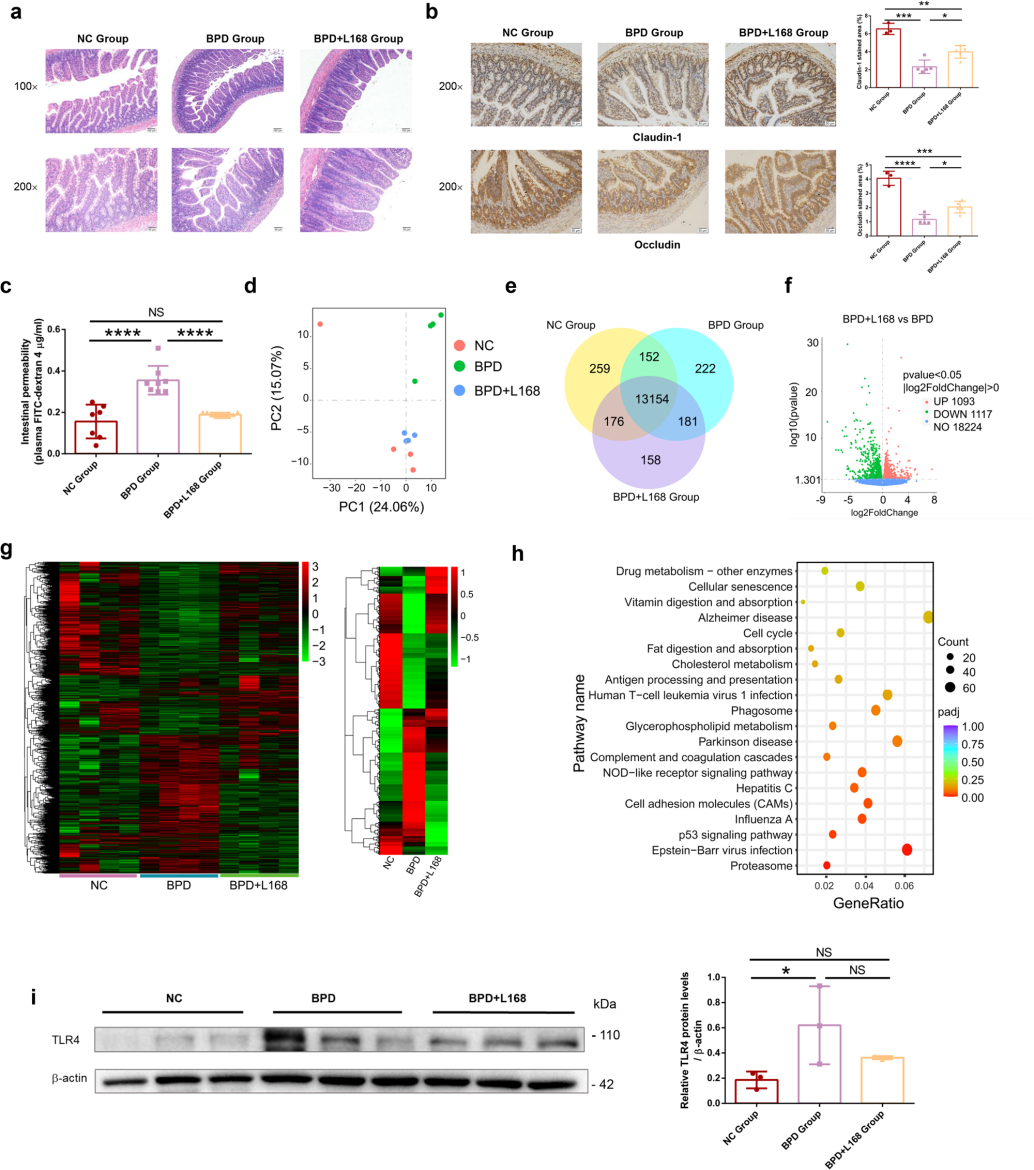
Oral *Lactobacillus plantarum* L168 improves barrier function and down-regulates inflammation-related gene of ileum in BPD. **a** Representative photomicrographs of HE-stained C sections (scale bar, 100 μm and 50 μm) (*n* = 3–5 each group). **b** Representative photomicrographs of immunohistochemical stained ileum sections for claudin-1 and occludin (scale bar, 50 μm) (*n* = 3–5 each group). **c** The plasma levels of FITC-dextran 4 (FD-4) after oral challenge in normal control, BPD and BPD + L168 rats (*n* = 7–9 each group). **d** Principal component analysis (PCA) of RNA sequencing of the ileum (*n* = 4 each group). **e** Co-expression Venn diagram among normal control, BPD and BPD + L168 rats. **f** The volcano plot of the differentially expressed genes between BPD and BPD + L168 rats. **g** The hierarchical clustering heatmap of differentially expressed genes among normal control, BPD and BPD + L168 rats. **h** Top 20 of KEGG pathway enrichment analysis of differentially expressed genes in BPD + L168 rats versus BPD rats. **i** The protein levels of TLR4 in the ileum by immunoblotting analysis. Data are expressed as the means ± SD (**b**, **c**, **i**) and one-way ANOVA was performed. ***p < 0.001; *p < 0.05; and NS not statistically significant.

To further understand the protective mechanism of *L. plantarum* L168 in gut-lung axis, we first assessed the gut barrier function of the rats. We performed HE staining of the ileum to check the inflammation and injury among groups (Fig. 4a). Among normal control rats, the intestinal villus structure was normal. However, in the untreated BPD group, the intestinal villi were swollen and shortened, the goblet cells were decreased, part of the epithelium was shed, the mucosal crypts were distorted, the interstitial blood vessels were dilated and congested, and chronic inflammatory cell infiltration was observed. Whereas treatment with *L. plantarum* L168 resulted in less inflammation and injury compared with the untreated BPD group. We also tested the immunohistochemical staining of claudin-1 and occludin (Fig. 4b). Compared with normal control rats, there was less area for claudin-1 and occludin in the untreated BPD rats, while *L. plantarum* L168 treatment elevated the area of both. We also examined intestinal permeability with FITC-dextran and found higher permeability of gut in the BPD rats compared with normal control rats. However, *L. plantarum* L168 treatment reduced the elevated permeability (Fig. 4c). Therefore, *L. plantarum* L168 played a vital role in improving the barrier function of gut in BPD rats.

### Oral administration of *Lactobacillus plantarum* L168 down-regulates inflammation-related genes of ileum in BPD rats

We next conducted RNA sequencing of the ileum to explore the underlaying potential mechanism of the protective role of *L. plantarum* L168 in gut function of BPD rats. From the principal component analysis (PCA) chart, the genes of the BPD untreated rats were significantly different from normal control and *L. plantarum* L168 treated rats (Fig. 4d). Co-expression Venn diagram also showed the co-expressed genes among the groups (Fig. 4e). Compared with BPD untreated rats, there were 2210 differentially expressed genes (DEGs) in *L. plantarum* L168 treated rats, with 1093 up-regulated and 1117 down-regulated genes (Fig. 4f). Heatmap of hierarchical clustering analysis (HCA) showed the crucial DEGs at the transcriptional level among the three groups (Fig. 4g). When compared to normal control rats, there were up-regulated and down-regulated differential genes in the BPD untreated rats. However, *L. plantarum* L168 treatment could partially reverse or improve the changes, revealing a beneficial regulatory effect on genes. We found that the DEGs associated with immunity and inflammation (e.g., genes that encode CCL2, CCL4, TLR4) decreased in the *L. plantarum* L168 treated group compared to BPD untreated group. Further, we analyzed the related pathway of DEGs based on KEGG database (Fig. 4h). From the perspective of pathway enrichment analysis, the most relevant pathways of DEGs altered by *L. plantarum* L168 were primarily involved in NOD-like receptor signaling, antigen presentation and expression, cell adhesion molecules, vitamin digestion and absorption, fat digestion and absorption, and other signaling pathways. Hyperoxia exposure significant increased the expression of TLR4 in the ileum, while *L. plantarum* L168 mitigated this increased trend, although the difference was not statistically significant (Fig. 4i). Therefore, *L. plantarum* L168 played an important role in down-regulating inflammation-related genes of ileum in BPD rats.

### Oral administration of *Lactobacillus plantarum* L168 regulates the metabolites of serum in BPD rats

**Fig. 5:**
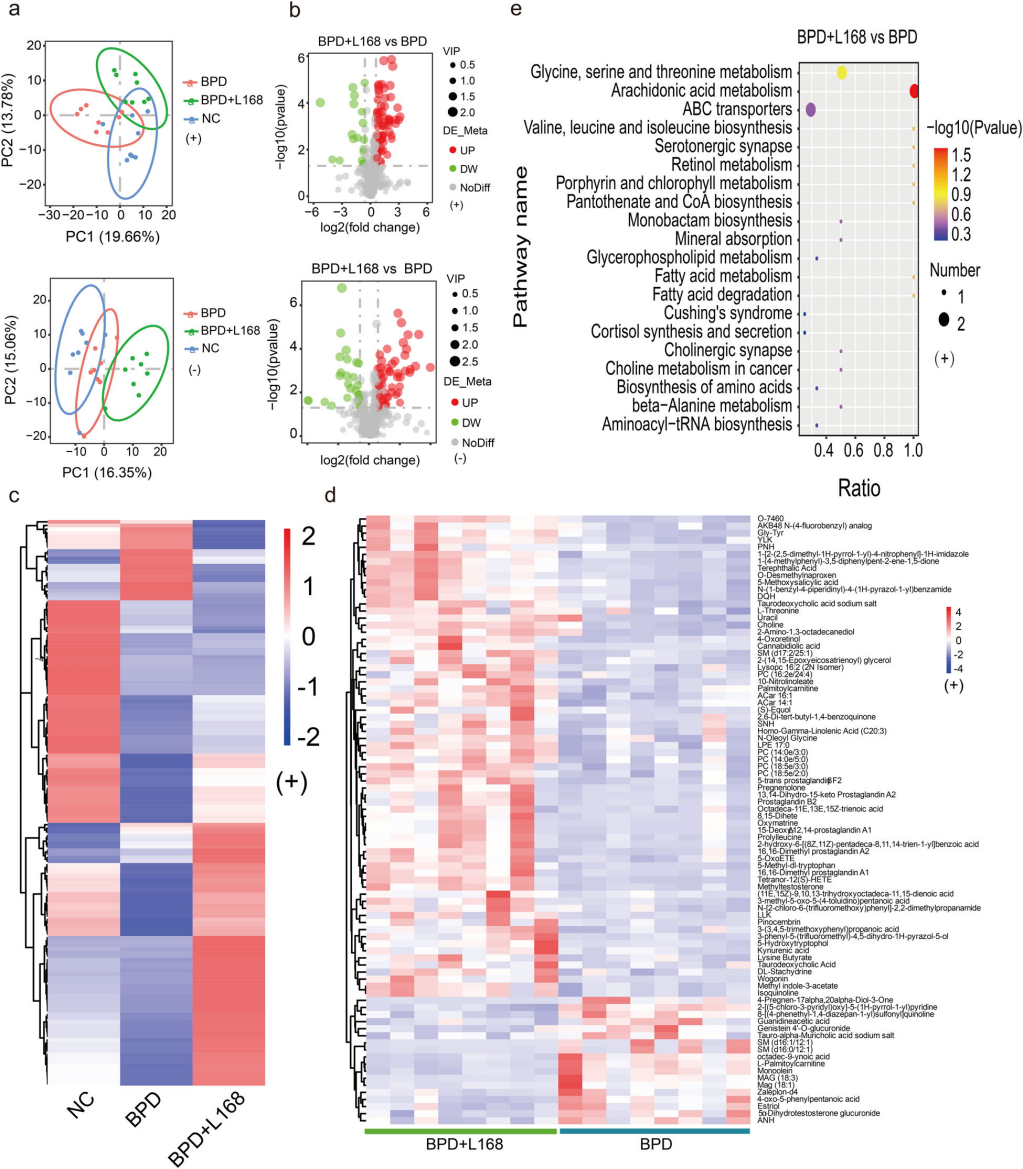
Oral *Lactobacillus plantarum* L168 regulates the metabolic state of circulation in BPD. **a** PCA of metabonomics in the serum among normal control, BPD and BPD + L168 rats: positive and negative ion modes (*n* = 8 each group). **b** The volcano plot of the differentially expressed metabolites between BPD and BPD + L168 rats: positive and negative ion modes. **c** The hierarchical clustering heatmap of differentially expressed metabolites among three groups: positive ion mode. **d** The hierarchical clustering heatmap of differentially expressed metabolites between BPD and BPD + L168 rats: positive ion mode. **e** Top 20 of KEGG pathway enrichment analysis of differentially expressed metabolites in BPD + L168 rats versus BPD rats: positive ion mode.

We then analyzed the serum metabolic profile of BPD untreated rats and *L. plantarum* L168 treated rats by LC-MS based metabolomics. Metabolites were qualitatively different among the groups according to the PCA chart (Fig. 5a). Differential expressed metabolites were shown by volcano plots, indicating the distinct metabolite levels between the BPD untreated and *L. plantarum* L168 treated rats (Fig. 5b). The PLS-DA chart showed the model was reliable and not overfitting (Supplementary Fig. 1a). The heatmap of HCA showed the major differential metabolites among the groups (Fig. 5c and Supplementary Fig. 1b), and there were up-regulated and down-regulated differential metabolites in the BPD untreated rats compared with the normal control rats. Encouragingly, some metabolites were reversed by *L. plantarum* L168, suggesting a beneficial regulatory effect on metabolites. Moreover, the metabolite levels in the BPD + L168 group were different from those in the BPD group through differential metabolite cluster analysis (Fig. 5d and Supplementary Fig. 1c). KEGG pathway enrichment analysis was further conducted for the differential metabolites. From the perspective of pathway enrichment analysis, the most relevant pathways of differential metabolites altered by *L. plantarum* L168 were primarily involved in biliary secretion, glutathione metabolism, arachidonic acid metabolism, serotonin synapses, fatty acid metabolism, and degradation (Fig. 5e and Supplementary Fig. 1d). Based on the results of blood metabolism, we pretreated mouse lung epithelial (MLE-12) cells with kynurenic acid and L-kynurenine before exposure to hyperoxia. The results showed protective effects on MLE-12 cells, ameliorating cell viability (Supplementary Fig. 2a–d) and attenuating cell apoptosis (Supplementary Fig. 2e, f). Taken together, we concluded that systematic metabolome was regulated by *L. plantarum* L168 administration, which may play a protective role in BPD.

### Oral administration of *Lactobacillus plantarum* L168 down-regulates inflammation-related genes of lung in BPD rats

**Fig. 6:**
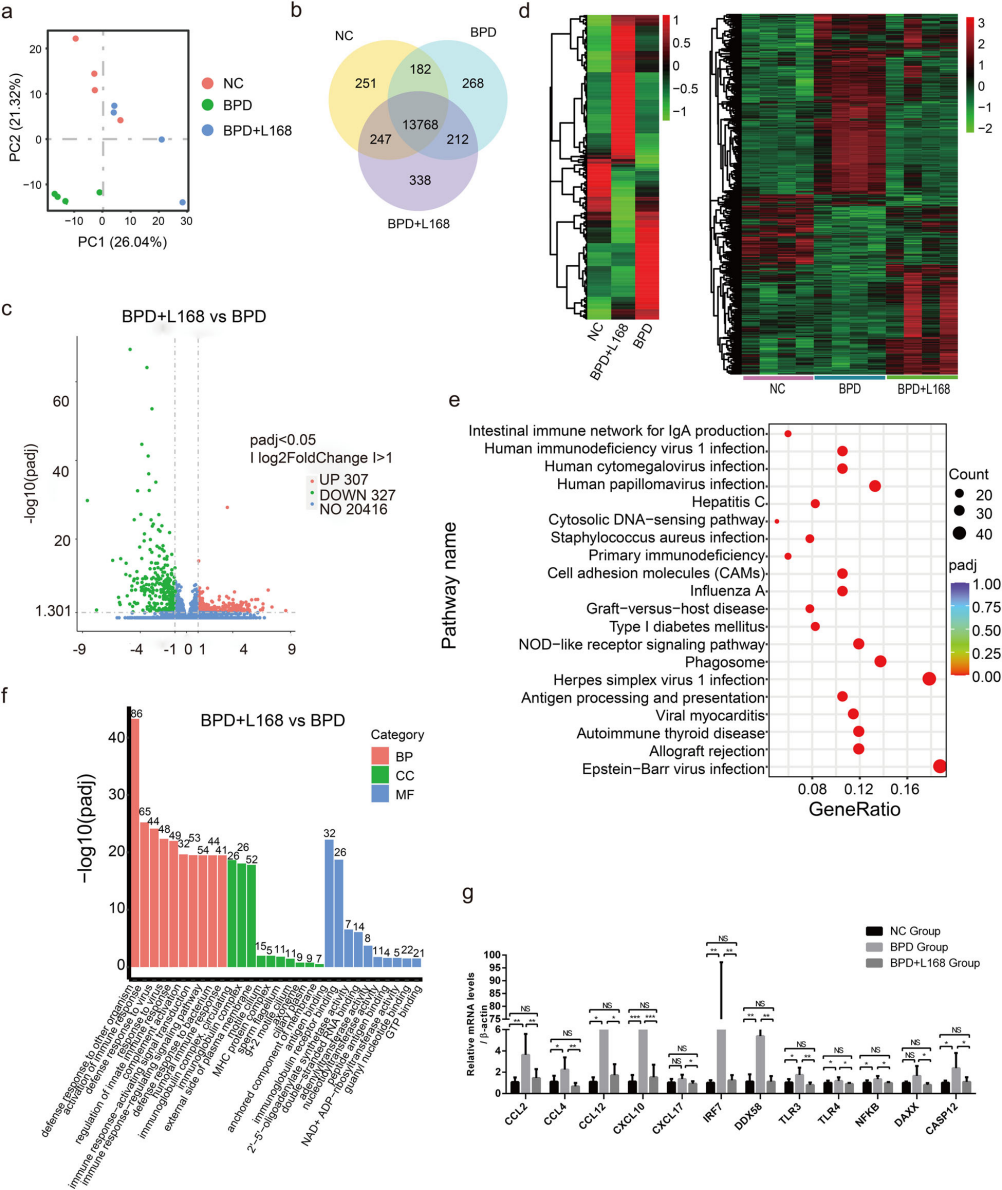
Oral *Lactobacillus plantarum* L168 down-regulates inflammation-related gene of lung in BPD. **a** PCA of RNA sequencing of the lung (*n* = 4 each group). **b** Co-expression Venn diagram among normal control, BPD and BPD + L168 rats. **c** The volcano plot of the differentially expressed genes between BPD and BPD + L168 rats. **d** The hierarchical clustering heatmap of differentially expressed genes among normal control, BPD and BPD + L168 rats. **e** Top 20 of KEGG pathway enrichment analysis of differentially expressed genes in BPD + L168 rats versus BPD rats. **f** Gene Ontology enrichment analysis of differentially expressed genes in BPD + L168 rats versus BPD rats: biological processes, cellular components and molecular functions. **g** Representative lung mRNA levels of genes associated with immunity and inflammation by RT-PCR analysis in normal control, BPD and BPD + L168 rats (*n* = 6 each group). Data are expressed as the means ± SD (**g**) and one-way ANOVA was performed. ***p < 0.001; *p < 0.05; and NS not statistically significant.

To address the underlying mechanism by which *L. plantarum* L168 improves lung injury and hypoalveolarization in BPD, we performed RNA sequencing of the lung tissues among groups. The PCA chart showed that the genes in the BPD untreated rats differed from normal control and *L. plantarum* L168 treated rats (Fig. 6a). Co-expression Venn diagram revealed the co-expressed genes among the groups (Fig. 6b). There were 634 DEGs between the BPD untreated rats and *L. plantarum* L168 treated rats, with 307 up-regulated and 327 down-regulated genes (Fig. 6c). The heatmap of HCA showed significantly changed genes at the transcriptional level of the lung among the three groups (Fig. 6d). Compared with normal control rats, we found there were up-regulated and down-regulated DEGs in the BPD untreated rats, while the *L. plantarum* L168 treatment could reverse or improve the changes in part, suggesting a protective regulatory effect on genes of *L. plantarum* L168. Further, KEGG pathway enrichment analysis of the DEGs showed that the top 20 KEGG pathways were mainly involved in NOD-like receptor signaling pathways, antigen presentation and expression, cell adhesion molecules, H1N1 influenza virus, phagosome signaling pathways and other signaling pathways (Fig. 6e). GO enrichment analysis showed the enriched GO terms (including biological process, cellular components, and molecular functions) in *L. plantarum* L168 treated rats were mainly related to the immune protective function, such as the protective response to other organs, activation of immune response, the response to virus and defense response, the activation of innate immune response, immune response activation signal transduction, immune response regulation signal transduction, immunoglobulin complex, antigen binding and immunoglobulin receptor binding (Fig. 6f). We also found that the expressions of genes associated with immunity and inflammation (e.g., genes that encode CCL2, CCL4, TLR4) were decreased in the *L. plantarum* L168 treatment rats compared with BPD untreated rats. Further, we confirmed the differences in gene expressions of the lung by RT-PCR, representing that CCL2, CCL4, CXCL10, and TLR4 were lower in the *L. plantarum* L168 treated rats compared with the BPD untreated rats (Fig. 6g). Therefore, we speculated that *L. plantarum* L168 down-regulated the expression of genes involved in immunity and inflammation, thus playing a protective role in BPD.

### *Lactobacillus plantarum* L168 treatment of BPD rats relates to down-regulation of the TLR4/NF-κB/CCL4 signaling pathway

**Fig. 7:**
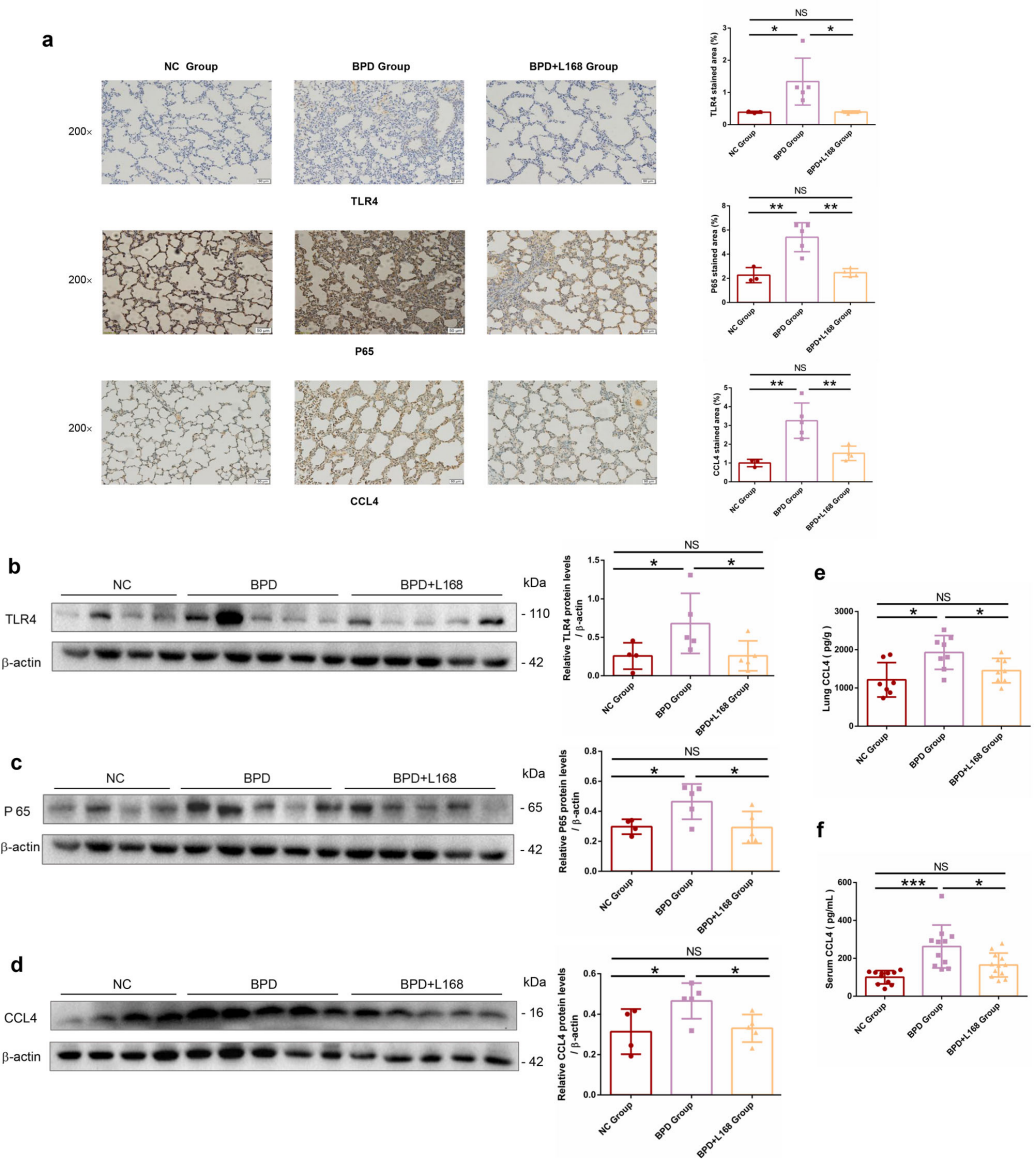
Oral *Lactobacillus plantarum* L168 improves BPD by down-regulating TLR4/NF-κB/CCL4 signaling pathway. **a** Representative photomicrographs of immunohistochemical stained lung sections for TLR4, P65 and CCL4 (scale bar, 50 μm) (*n* = 3–5 each group). **b**–**d** The protein levels of TLR4, P65 and CCL4 in the lung by immunoblotting analysis (*n* = 4–5 each group). **e** CCL4 level in the lung in normal control, BPD and BPD + L168 rats (*n* = 7–8 each group). **f** The serum level of CCL4 in normal control, BPD and BPD + L168 rats (*n* = 10–12 each group). Data are expressed as the means ± SD (**a**–**f**) and one-way ANOVA was performed. ***p < 0.001; *p < 0.05; and NS not statistically significant.

KEGG signaling pathway analysis of DEGs in lung transcriptomics revealed that the Toll-like receptor signaling pathway was down-regulated after *L. plantarum* L168 treatment (Supplementary Fig. 3). Among the genes involved in the pathway, TLR1, TLR4, TLR7, TLR8, MIP-1β (CCL4), and IL-10 were down-regulated. Immunohistochemical analysis confirmed significant up-regulation of TLR4, P65 and CCL4 expressions in BPD lung tissues. However, treatment with *L. plantarum* L168 down-regulated their expression levels, showing no significant difference compared to the normal control group (Fig. 7a). Similar results were confirmed by Western blotting (Fig. 7b–d). ELISA was also used to detect the expression of CCL4 in serum and lung tissue, which also showed that *L. plantarum* L168 significantly down-regulated the expression of CCL4 (Fig. 7e, f). Previous studies implicated that overactivation of TLR4 and NF-κB impaired lung function and aggregated lung inflammation^26–29^. Therefore, we deduced that *L. plantarum* L168 could improve BPD relating to down-regulation of the TLR4 /NF-κB /CCL4 signaling pathway.

## Discussion

**Fig. 8:**
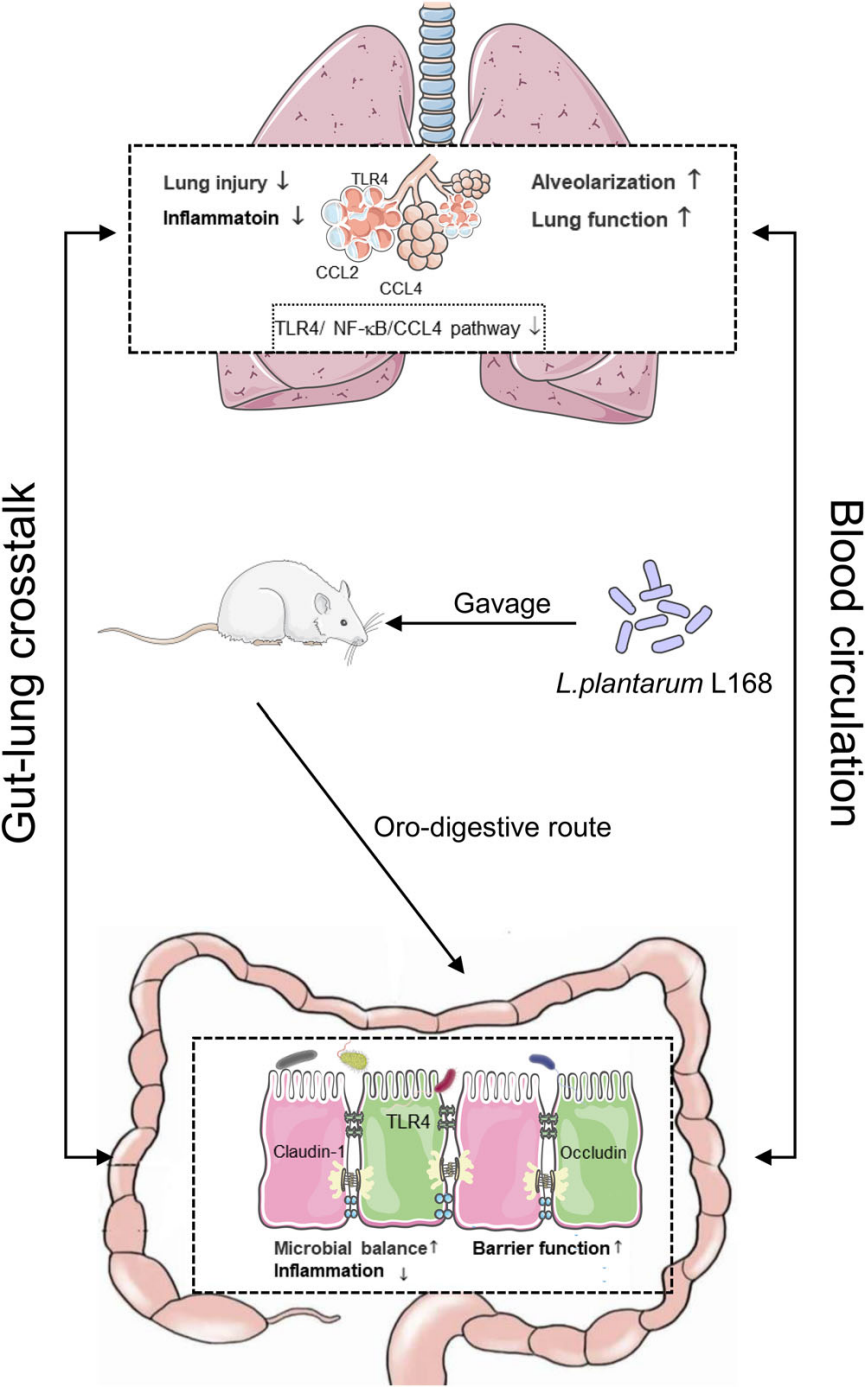
Working model of the schematic diagram of *Lactobacillus plantarum* L168 protection against hyperoxia-induced pulmonary inflammation and hypoalveolarization in bronchopulmonary dysplasia. *L. plantarum* L168 improves the intestinal mucosal barrier, restores the imbalance of metabolism, and reduces the inflammatory response through the gut-lung axis.

In our study, as shown in Fig. 8, we first found the dysbiosis of gut microbiota in preterm infants and neonatal rats with BPD, suggesting a possible link between gut microbiota and BPD. We revealed that a probiotic, *L. plantarum* L168 could improve the short term prognosis of BPD rats, thereby exerting a beneficial protective effect for BPD rats. The multi-omics analysis showed that *L. plantarum* L168 could ameliorate BPD predominantly by improving the intestinal mucosal barrier, restoring of imbalance of metabolism, and reducing the inflammatory response.

In preterm infants, research on the microbiome has initially focused on the relationship between the gut microbiota and necrotizing enterocolitis^30,31^. With the development of the gut-lung axis hypothesis, more and more studies have linked gut microbiota dysbiosis to the development of BPD. In the neonatal intensive care unit, preterm infants are usually exposed to oxygen or ventilation after birth due to respiratory distress syndrome or the weakness of whole body. Nevertheless, hyperoxia exposure was reported to cause gut dysbiosis, affect mucosal immunity and impair intestinal mucosal barrier^32–35^. Our study on the gut microbiota in BPD premature infants and BPD rats found a decrease in Firmicutes and an increase in Bacteroidetes, which is consistent with the trend of dysbiosis in the microbiota of lung tissue in BPD susceptible infants reported by Lal et al.^36^. In the current study, we also verified that the intestinal barrier function was weakened and inflammatory infiltration was increased in BPD rats, which is consistent with previous reports^32^. Encouragingly, *L. plantarum* L168 alleviated intestinal inflammation, repaired intestinal epithelial tight junctions, and improved barrier function. Overall, the results suggested that the dysbiosis of gut microbiota in BPD might influence the immune and inflammatory state of the lung through the gut-lung axis^32^.

Microbiota plays a crucial role in the immune, hormonal and metabolic homeostasis of its host. *Lactobacillus* has shown strong anti-inflammatory properties. Notably, we observed that *Lactobacillus* was significantly declined in the gut microbiota of BPD rats, and the change was likely related to the excessive inflammatory response in BPD. Further, we carried out the intervention of *L. plantarum* L168 in BPD rats and observed significant improvement in the lung function. To further explore BPD-related metabolic pathways based on gut microbial patterns, we performed an untargeted metabolomic analysis and found that *L. plantarum* L168 can improve BPD-related metabolic disorders. Previous studies indicated that gut microbiota could affect the metabolic state by producing metabolites, inflammatory mediators, and so on^32^. Further, KEGG pathway enrichment analysis indicated that the pathways involved were mainly related to the synthesis and metabolism of amino acids, the metabolism and degradation of fatty acids, and the metabolism of arachidonic acid, linoleic acid, and glutathione.

Interestingly, kynurenic acid and 5-hydroxyindoleacetic acid in the tryptophan metabolic pathway in the BPD group significantly declined compared with normal control group, while the two metabolites up-regulated after the intervention of *L. plantarum* L168. Both kynurenic acid and 5-hydroxyindoleacetic acid are endogenous metabolites of tryptophan, which have been shown to prevent amyloid-β peptide-induced neurotoxicity, thereby alleviating Alzheimer’s disease, and are considered as neuroprotective agents^37^. In addition, kynurenic acid has also been shown to promote the expression of anti-inflammatory genes and reduce the expression of cellular markers and cytokines associated with inflammation or type I immune responses^38^. Our cell experiments similarly suggested the protective effects of kynurenic acid and L-kynurenine in hyperoxia-exposed MLE-12 cells. Consistent with this, in our study, *L. plantarum* L168 was found to significantly upregulate the level of human gamma-linolenic acid. Encouragingly, human gamma-linolenic acid was reported to have a tumor-inhibiting effect^39^ by reducing soluble intercellular adhesion molecule-1 and monocyte chemotactic protein, and regulating the activation, expression, and secretion of normal T cells^40^. In the present study, *L. plantarum* L168 also upregulated prostaglandin A1 (PGA1) and A2 (PGA2) levels. PGA1 was reported to cause dysregulation of influenza virus A protein synthesis, prevention of virus-induced disassembling of the host microtubule network, and activation of the proinflammatory factor NF-κB^41^. PGA2 protected the endothelial cell barrier and inhibited LPS-induced inflammatory signaling, thereby exerting a protective role in both LPS and mechanical ventilation-induced acute lung injury^42^. Taken together, the metabolism assay implicated that *L. plantarum L168* could regulate the circulating metabolic state, reduce the expression of pro-inflammatory factors and stimulate the expression of anti-inflammatory factors. Therefore, we speculated that *L. plantarum* L168 might regulate the metabolic state of the body by restoring the ecological balance of the gut microbiota, and affect the immune and inflammatory responses of the systemic and local target organs through metabolites, thereby protecting against BPD.

Currently, it is believed that excessive secretion of pro-inflammatory cytokines under pathological factors contributes greatly to BPD, including resulting in persistent inflammation, inhibiting lung’s secondary septation, alveolarization and normal vascular development, and even altering the ability to repair^1,3^. Recently, Pietrzyk et al. reported that BPD patients have nearly 10% variation in gene expression associated with inflammatory responses^43^. Hirani et al. found that the gene expressions of macrophage-regulating chemokines, such as CCL2, CCL7, CXCL5, etc, were over-expressed in BPD newborn mice induced by hyperoxia^44^. In agreement with these results, we found that the expressions of chemokines and inflammatory/apoptosis-related genes, such as CCL2, CCL4, TLR4, etc. were up-regulated in BPD rats. Interestingly, *L. plantarum* L168 significantly inhibited the up-regulation of related genes above. KEGG pathway enrichment analysis also showed that the changed genes were involved in the innate immune response, the response to bacteria, Toll-like receptor (TLR) signaling pathways, the nucleotide oligomerization domain (NOD)-like receptor (NLR) signaling pathways, the retinoic acid-induced Gene I (RIG-I)-like receptor (RLR) signaling pathways, cytokine-cytokine receptor interaction, and chemokine signaling pathways.

TLRs, NLRs, and RLRs, which belong to pattern recognition receptors^45^, are the first line of self-defense for hosts as regulators of the innate immune system. When the endogenous danger signals are recognized, TLRs are first up-regulated to initiate the innate immune responses. However, excessive or inappropriate immune responses may cause inflammatory damage^46^. Previous studies have shown that activation of TLR4 inhibits the development of distal airway branching and alveolarization in fetal rat lungs^26^. The activation of TLRs and the release of inflammatory mediators also inhibited the expression of key developmental genes in mesenchymal cells of fetal lung, indicating that excessive activation of TLRs is one of the major mechanisms in the pathogenesis of BPD^27,47^. In our study, the KEGG pathway analysis of the lung transcriptome data demonstrated that the TLR4 expression was significantly down-regulated after the intervention of *L. plantarum* L168, which was associated with a significant decrease in the inflammatory chemokine MIP-1β (CCL4). This is consistent with the known beneficial effects of blocking or down-regulating the expression of TLR4 and NF-κB in BPD^28,29^. Taken together, our findings suggested that *L. plantarum* L168 may provide therapeutic benefits for patients with BPD in clinical practice.

Our study has a few limitations. The human sample size was relatively small, and we did not conduct a classification and stratification analysis of the antibiotics used. Multicenter and more extensive clinical studies are needed to further explore the dysbiosis of gut microbiota in BPD preterm infants. Some studies showed that male sex is a risk factor for BPD^3,48–50^. Therefore, in order to eliminate the confounding effect of mixed gender on the BPD model, we used male rats only. It is necessary to conduct study for female rats in the future. Although our multi-omics analysis comprehensively prompted the potential contribution of *L. plantarum* L168 to BPD rats, more indepth research is needed to further investigate how the gut-lung axis is influenced by *L. plantarum* L168.

In conclusion, we found the dysbiosis of gut microbiota in preterm infants and neonatal rats with BPD, suggesting a possible link between gut microbiota and BPD. *L. plantarum* L168 supplementation reduced lung injury and improved lung development of BPD rats. After treatment with *L. plantarum* L168, rats had significant increases in serum metabolites with anti-inflammatory effects. In agreement with the metabolites analysis of serum, RNA-seq analysis of the intestine and lung discovered that inflammation and immune-related genes were significantly down-regulated. Furthermore, we validated that *L. plantarum* L168 might improve BPD relating to down-regulation of TLR4/NF-κB/CCL4 signaling pathway by Western blotting and immunohistochemistry staining. Together, these findings suggested that *L. plantarum* L168 might improve inflammation and hypoalveolarization in BPD rats through the gut-lung axis, highlighting its potential as a probiotic-based therapeutic approach for BPD. Further studies should focus on the underlying mechanism of the gut-lung axis being regulated by the probiotics.

## Methods

### Patients

A total of 15 BPD premature infants and 18 non-BPD controls were included in this study. All premature infants were recruited from the Children’s Hospital of Nanjing Medical University. The diagnosis of BPD status was assessed by the definition of the National Institute of Health (NIH) workshop^51^. BPD was defined as oxygen requirement >21% for at least 28 days. The severity of BPD was evaluated at 36 weeks post-menstrual age or discharge (whichever comes first) according to the level of respiratory support needed: Mild: breathing room air, Moderate: need for 30% O_2_, Severe: need for >30% O_2_, and/or positive pressure (nasal continuous positive airway pressure or positive-pressure ventilation). Premature infants with congenital sepsis, evidence of congenital airway or pulmonary hypoplasia, and major congenital anomalies were excluded. Feces samples were collected at 28–30 days post menstrual age. In depth clinical information such as hospital course, birth history and maternal history were collected. The clinical study was approved by the Ethics Committee of the Children’s Hospital of Nanjing Medical University and conceded waiver of consent status as samples were collected through routine clinical care (No. 202110094-1).

### Animals

The Sprague-Dawley rats pregnant at E14-E16 were ordered from Weitong Lihua Lab Animal Tec. Co. Ltd. (Zhejiang, China) and housed in the experimental animal center of Nanjing Medical University based Specific Pathogen Free facility. All rats were kept at a typical 12–12 h cycle of light/dark and an average temperature of 22 °C. Male neonatal rats were utilized for the current study. Animal experiments were approved and supervised by the Animal Management Committee of Nanjing Medical University (No. IACUC-2107039).

### BPD rats model establishment

Based on the well-known and established model of BPD^25^, neonatal rats with their mothers were randomly assigned to two groups within 24 h after birth: one group kept in normoxia (room air, FiO_2_ 21%) and the other group exposed to hyperoxia (FiO_2_ 75–80%). A big plexiglas chamber was used to conduct the hyperoxia experiment, and the rats of hyperoxia group were kept in it. Oxygen was delivered into the chamber continuously to achieve a level of 75–80% O_2_, through an oxygen generator. In order to remove the excess CO_2_ and water vapor, soda lime was placed in the chamber. Rats exposed to hyperoxia were taken out of the chamber for half an hour every 24 h, to prevent oxygen toxicity in the mothers. The two groups were kept in room air or hyperoxia for the same duration of time (until 2 weeks after birth). Rats were euthanized by inhaled isoflurane following approved ethical guidelines, and then harvest was carried out. There were at least 10 male neonatal rats from 3 individual litters in each group.

### BPD rats treatment

After 2 weeks exposure to hyperoxia, the BPD model was established. On the 15th day after birth, the BPD rats were returned to room air and immediately divided into two groups: the BPD + L168 group and the BPD control group. The BPD + L168 group was given *L. plantarum* L168 (10^8^ cfu/ml, 0.1 ml/10 g) by gavage once a day. The BPD control group was given phosphate buffer saline (PBS) (0.1 ml/10 g) by gavage daily. The normal control neonatal rats were also given PBS (0.1 ml/10 g) by gavage once a day. All treatments lasted for 2 weeks.

### Respiratory data establishment

Respiratory data were analyzed by an animal pulmonary function testing system (Beilanbo Technology Co., LTD, Beijing, China) after 2 weeks of treatment. After satisfactory anesthesia by inhaled isoflurane, the rats were tracheotomized, intubated, and then placed inside a closed Whole Body Plethysmograph connected to a transducer and a computer. We evaluated the resistance of lung (R) and respiratory dynamic compliance (C).

### Gut permeability analysis

Gut permeability was assessed by measuring the fluorescein isothiocyanate conjugated-dextran (FD-4) levels of plasma. First, we used PBS (concentration of 50 mg/ml) to diffuse FITC-dextran and treated each rat with FD-4 (gavage, 0.1 ml/10 g) 4 h prior to euthanasia. Plasma was then harvested, and serum was isolated following the manufacturer’s instructions. Serum was mixed with PBS (equal volume) and then added to a microplate (96 wells) in duplicate. The Spectro photo-fluorometry (BioTek, USA) was used to measure FD-4 concentration in serum (485 nm excitation and 528 nm emission wavelength). The serially diluted samples were taken as standards, and all the detected samples were analyzed in triplicate.

### Tissue processing and histological analysis

The lung and ileum tissue samples were placed in paraffin-embedded paraformaldehyde (4%) and then cut into thick sections (3–4 µm) for histological analysis and immunohistochemistry test. Hematoxylin and eosin (HE) was performed to estimate the histomorphologic feature. Sirius Red and Masson’s Trichrome stains were performed to evaluate fibrosis of the lung. For immunohistochemical analysis, primary antibodies included Claudin-1 (Servicebio, 1:200), Occludin (Servicebio, 1:200), TLR (Servicebio, 1:500), P65 (Servicebio, 1:800), and CCL4 (ABclonal, 1:100). Olympus BX51 microscope (Tokyo, Japan) was used for histological examination.

### Cytokine and chemokine measurements

The rats’ serum levels of tumor necrosis factor α (TNF-α), Macrophage inflammatory protein-1β (MIP-1β, CCL4) and interleukin-6 (IL-6) were evaluated using enzyme linked immunosorbent assay (ELISA) kits (Elabscience, China) following to the manufacturer’s guidelines. CCL4 was detected in whole lung homogenate following the manufacturer’s instructions by the ELISA kits (Elabscience, China).

### Quantitative real-time PCR analysis

Total RNA of tissue samples was isolated, added TRIzol, and then reverse-transcribed to obtain cDNA. Quantitative reverse transcription-PCR (Roche, Switzerland) was conducted with an SYBR Green PCR Master Mix (Vazyme, China). Expression of genes was quantified by the ΔΔCT method and used β-actin as a reference gene. The primers applied in our study are listed (Table 2).

**Table 2:** Primers for RT-PCR

Gene name	primer sequence (5ʼ-3ʼ)	
Rat-β-actin	F	5ʼ-CACCATGTACCCAGGCATTG-3ʼ
	R	5ʼ-CCTGCTTGCTGATCCACATC-3ʼ
Rat-CCL12	F	5ʼ-TTGGCTGGACCAGATTCAGT-3ʼ
	R	5ʼ-GACACTGGCTGCTTGTGATT-3ʼ
Rat-CCL 2	F	5ʼ-CAGCCAACTCTCACTGAAGC-3ʼ
	R	5ʼ-GTGAACAACAGGCCCAGAAG-3ʼ
Rat-CCL 4	F	5ʼ-CAGCTCTGTGCAAACCTCTC-3ʼ
	R	5ʼ-GGAGGAGAGAGAAGGCAGAC-3ʼ
Rat-CXCL10	F	5ʼ-TCCTGTCCGCATGTTGAGAT-3ʼ
	R	5ʼ-GCGAGTGGCTTCTCTCTAGT-3ʼ
Rat-CXCL17	F	5ʼ-GCCTCTCCCTTCCTTCTGTT-3ʼ
	R	5ʼ-CTCGCAGGGACCAATCTTTG-3ʼ
Rat-TLR 3	F	5ʼ-CTCCCGCACCTGAAGTATCT-3ʼ
	R	5ʼ-GGTGAGAAGCAAGTGCAACA-3ʼ
Rat-TLR4	F	5ʼ-TAGCCATTGCTGCCAACATC-3ʼ
	R	5ʼ-ACACCAACGGCTCTGGATAA-3ʼ
Rat-IRF 7	F	5ʼ-CCTCTGCTTTCTGGTGATGC-3ʼ
	R	5ʼ-GCTCCTCAGGAAGGTGTTCT-3ʼ
Rat-DDX58	F	5ʼ-CTGATGAGGGAGGCAGAGAG-3ʼ
	R	5ʼ-GAAACACCGTGCATGCTTTG-3ʼ
Rat-DAXX	F	5ʼ-TCAATGGGCGTGTCTCTTCT-3ʼ
	R	5ʼ-ATCTTCCACCCACTGTCCTG-3ʼ
Rat-CASP 12	F	5ʼ-TTTATGTCCCACGGCATCCT-3ʼ
	R	5ʼ-GGCTATCCCTTTGCTTGTGG-3ʼ
Rat-NFKBia	F	5ʼ-TTGGTCAGGTGAAGGGAGAC-3ʼ
	R	5ʼ-GGATCACAGCCAGCTTTCAG-3ʼ
Rat-Occludin	F	5ʼ-AAACCGACTACACGACAGGT-3ʼ
	R	5ʼ-TCCAGTTCTCTGTCGAGACG-3ʼ
Rat-Claudin 1	F	5ʼ-CTACGGAGGGTACACAGACC-3ʼ
	R	5ʼ-CACCATGATGCCCAGGATTG-3ʼ

### Western blotting

Protein of lung and ileum was extracted and placed in a Radio-immunoprecipitation assay (RIPA) buffer with added protease and phosphate inhibitors. Immunoblotting was conducted with primary antibodies against TLR4 (ABclonal, A5258, 1:1000), P65 (Cell Signaling Technology, 8242S, 1:1000), CCL4 (ABclonal, A1671, 1:1000) and β-Actin (ABclonal, AC038, 1:10000), and then resuspended with HRP Goat Anti-Rabbit IgG secondary antibody (ABclonal, AS014, 1:5000). Finally, the blottings were exposed to X-ray films with the Enhanced Chemiluminescence system (BioRad, Hercules, CA, USA). All blots or gels derive from the same experiment and they were processed in parallel. Original blots are provided in Supplementary Materials.

### Cell culture and treatment

Mouse lung epithelial (MLE-12) cells were obtained from the Cell Bank of the Typical Culture Collection Committee of the Chinese Academy of Sciences. MLE-12 cells were cultured in DMEM/F12 medium (Gibco) containing 10% fetal bovine serum (FBS, Gibco), 100 ml streptomycin/penicillin, and maintained at 37 °C and 5% CO_2_ in a humidified incubator. For subsequent assay, MLE-12 cells were randomly divided into four groups: control, hyperoxia, hyperoxia + kynurenic acid and hyperoxia + L-kynurenine. The control group was placed in the humidified incubator with 37 °C and 5% CO_2_. The other three groups were placed in an oxygen container with 80–85% O_2_, 37 °C and 5% CO_2_ for culture.

### Cell proliferation assay

The viability of MLE-12 cells was measured by the CCK-8 assay kit (Beyotime, cat: C0042). MLE-12 cells were cultured in 96-well plate with DMEM/F12 medium and treated with kynurenic acid (400, 800, 1600, 3200 nM) or L-kynurenine (50, 100, 200, 400 μM) for 48 h. After washing with PBS, cells were treated with 100 μl DMEM medium containing 10 μl CCK-8 solution for another 2 h at 37 °C. The values were measured by a microplate reader at 450 nm (BioTek Instruments, Inc., Winooski, VT, United States).

### Cell apoptosis analysis

Cell apoptosis was detected by the Annexin V-FITC apoptosis detection Kit (Beyotime, cat: C1062L). MLE-12 cells were cultured in 12-well plate with DMEM/F12 medium and treated with kynurenic acid (400, 800 nM) or L-kynurenine (100, 200 μM) for 48 h. Cells were harvested for double staining with Annexin V-FITC and propidium iodide (PI) solution according to the instruction. The cell apoptosis was detected with a flow cytometer (Beckman coulter, CytoFLEX) and the results were analyzed by CytExpert software.

### Fecal microbial DNA isolation and gene sequencing of 16S rRNA

Microbial genomic DNA was isolated from fecal samples according to the protocols of a Stool DNA extraction kit (DP328, Tiangen, Beijing, China). OE Biotech (Co., Ltd. Shanghai, China) carried out the 16S ribosomal RNA (rRNA) analysis. In 50 µl triplicate solutions, genomic DNA was amplified using bacterial 16S rRNA gene V3-V4 region-specific primers: 338F (5’-TACGGRAGGCAGCAG-3’) and 806R (5’-AGGGTATCTAATCCT-3’). Reverse primer was labeled with a sample barcode, and both primers were linked to a sequencing adapter of Illumina. Cleaned PCR products were then adjusted in concentration for sequencing on the system of Illumina Miseq PE300 equipment (Illumina, USA). Reads of original sequencing were classified with specific barcodes, and then the PCR primer sequences, linker, and barcode were discarded. The quality criteria fulfilled sequences were examined and designated for bioinformatics assessment. QIIME 2 software was used for the 16S rRNA sequencing analysis^52^.

### Untargeted metabolomics analysis

An aliquot of 100 μl of serum was placed in an Eppendorf tube and then methanol (80%, prechilled) was added. After 5 min on ice, the samples were centrifuged at 15,000 × *g* for 20 min at 4 °C. Liquid Chromatography-Mass Spectrometry (LC-MS) grade water was used to dilute some of the supernatants to a final concentration with methanol (53%). The supernatant was loaded into the LC-MS/MS system and analyzed by Novogene (Co., Ltd. Beijing, China) utilizing an Orbitrap Q Exactive TMHF-X mass spectrometer (Thermo Fisher, Germany) paired with a Vanquish UHPLC system (Thermo Fisher, Germany) after centrifugation at 15,000 × *g* for 20 min at 4 °C. With the use of the KEGG database (https://www.genome.jp/kegg/pathway.html), HMDB database (https://hmdb.ca/metabolites) and LIPIDMaps database (http://www.lipidmaps.org/), metabolites were annotated. The statistically significant difference (*p* value) was determined using univariate analysis (*t*-test). In order to qualify as differential metabolites between groups, the metabolite has to have a VIP > 1, a *p* value < 0.05, a fold change ≥2, or an FC ≤ 0.5. The KEGG database was utilized to investigate the roles of various metabolites and metabolic pathways.

### Transcriptome sequencing and gene expression analysis

RNA sequencing (RNA-Seq) and analysis were performed by Novogene company (Beijing, China). The Agilent Bioanalyzer (2100 System, Agilent Tech., CA, USA) was used to assess the quality of the generated libraries. Following that, Illumina NovaSeq (6000, Illumina, USA) was used to sequence the qualifying libraries, producing 150 bp paired end reads. Clean reads were extracted under quality control from the raw data by deleting reads containing adapter, reads 1 carrying ploy-N, and low-quality reads before being matched to the reference genome using Hisat2 (v2.0.5). The fragments per kilobase per million mapped reads (FPKM) was utilized to calculate gene expression. The DESeq2 R software (1.20.0) was applied to evaluate the DEGs. In order to reduce the false discovery rate, the obtained *p* values were modified using Benjamini and Hochberg’s method. DESeq2 identified DEGs as those having an adjusted *p* value < 0.05. Gene ontology (GO) enrichment analysis and Kyoto Encyclopedia of Genes and Genomes (KEGG) pathways were carried out in order to further investigate the DEGs. Significant enrichment of DEGs was defined as GO pathways with a corrected *p* value < 0.05. To examine the statistical enrichment of DEGs in KEGG pathways, the cluster profile R tool was employed.

### Statistical analysis

SPSS23.0 software was used for the statistical analysis. Data conformed to normal distribution were presented as mean ± SD, while non-normally distributed data were expressed as median (interquartile range). Measurement data with normal distribution were compared between two groups using two independent sample *t*-tests, and multiple groups were compared using ANOVA. Non-parametric test (Mann–Whitney *U* test) was used for the comparison of measurement data with non-normal distribution between two groups. Chi-square test was used for enumeration data. *p* < 0.05 indicates the differences were statistically significant.

### Reporting summary

Further information on research design is available in the Nature Research Reporting Summary linked to this article.

### Supplementary information

Supplementary Figures

Reporting Summary

## Data Availability

All 16S rRNA and RNA-seq raw data have been deposited into NCBI database under accession code PRJNA895707, PRJNA895708, PRJNA899514 and PRJNA899515. All data are available from the corresponding authors upon reasonable request.
