# Author Correction: *Lactobacillus plantarum* L168 improves hyperoxia-induced pulmonary inflammation and hypoalveolarization in a rat model of bronchopulmonary dysplasia

**DOI:** 10.1038/s41522-024-00516-6

**Published:** 2024-05-10

**Authors:** Xian Shen, Zhaocong Yang, Qiang Wang, Xu Chen, Qihui Zhu, Zhi Liu, Nishant Patel, Xingyin Liu, Xuming Mo

**Affiliations:** 1 Department of Neonatology, Children’s Hospital of Nanjing Medical University, Nanjing, China; 2 Department of Cardiothoracic Surgery, Children’s Hospital of Nanjing Medical University, Nanjing, China; 3 State Key Laboratory of Reproductive Medicine, Key Laboratory of Pathogen of Jiangsu Province, Key Laboratory of Human Functional Genomics of Jiangsu Province Center of Global Health, Nanjing Medical University, Nanjing, China

**Keywords:** Applied microbiology, Clinical microbiology

Correction to: *npj Biofilms and Microbiomes* 10.1038/s41522-024-00504-w, published online 29 March 2024

In the original version of this Article, the funding from the National Key Research and Development Program (2022YFA1303900) was omitted by mistake. It has now been added to the HTML and PDF versions of the Article.

