# Supplementary material for: Lactobacillus plantarum L168 improves hyperoxia-induced pulmonary inflammation and hypoalveolarization in a rat model of bronchopulmonary dysplasia
Source: NPJ Biofilms Microbiomes. 2024 Mar 29;10:32. doi: 10.1038/s41522-024-00504-w (PMC10980738; doi:10.1038/s41522-024-00504-w)

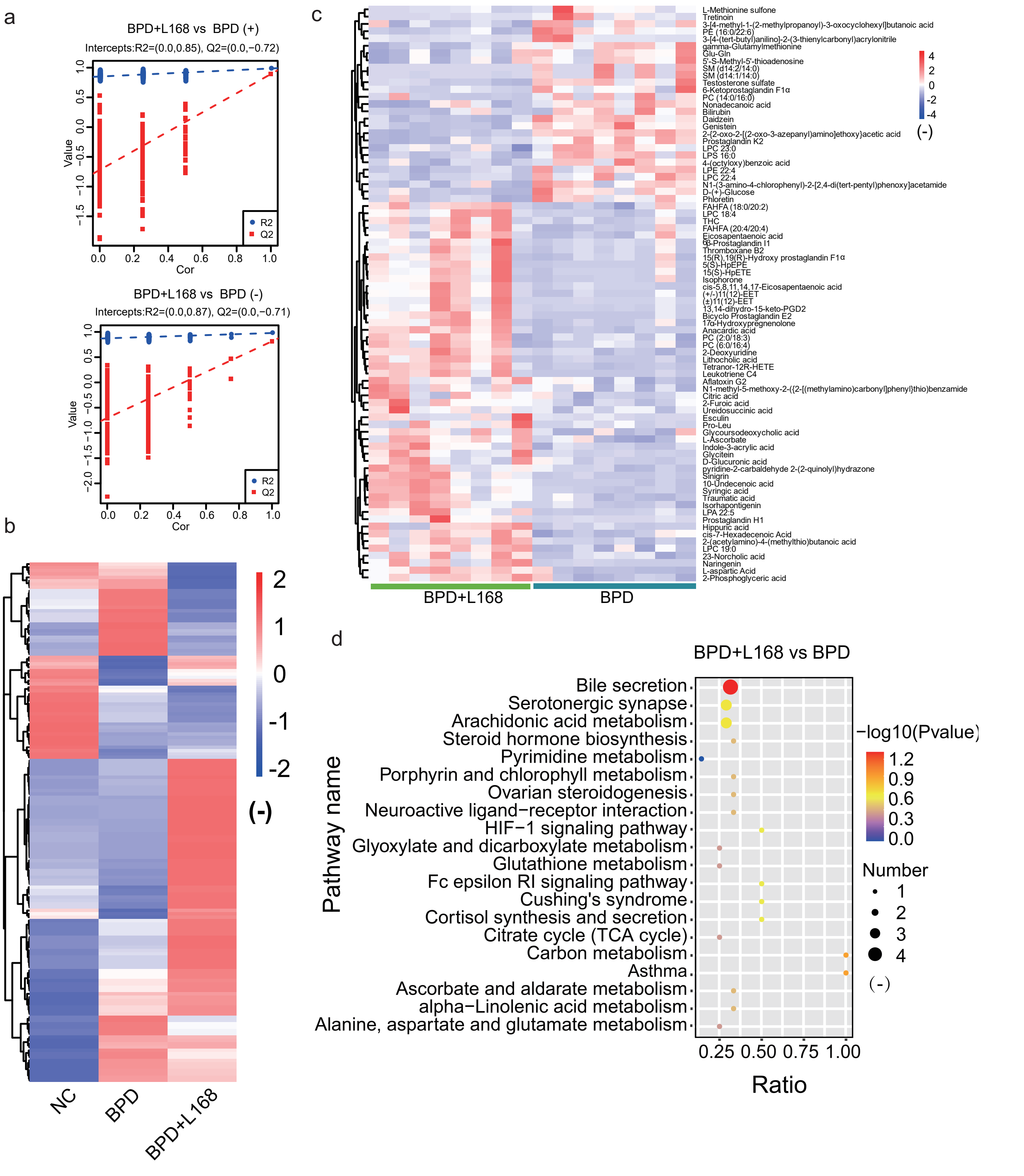

Supplementary Figure 1. Oral Lactobacillus plantarum L168 regulates the metabolic state of circulation in BPD. (**a**) Statistical validation of the PLS-DA model by permutation testing: positive and negative ion modes (n=8 each group).The intercepts of R2 = (0.0, 0.85) and Q2 = (0.0,–0.72), R2 = (0.0, 0.87) and Q2 = (0.0,–0.71) indicate that the PLS-DA models are not overfitting. (**b**) The hierarchical clustering heatmap of differentially expressed metabolites among three groups: negative ion mode. (**c**) The hierarchical clustering heatmap of differentially expressed metabolites between BPD and BPD+L168 rats: negative ion mode. (**d**) Top 20 of KEGG pathway enrichment analysis of differentially expressed metabolites in BPD+L168 rats versus BPD rats: negative ion mode.

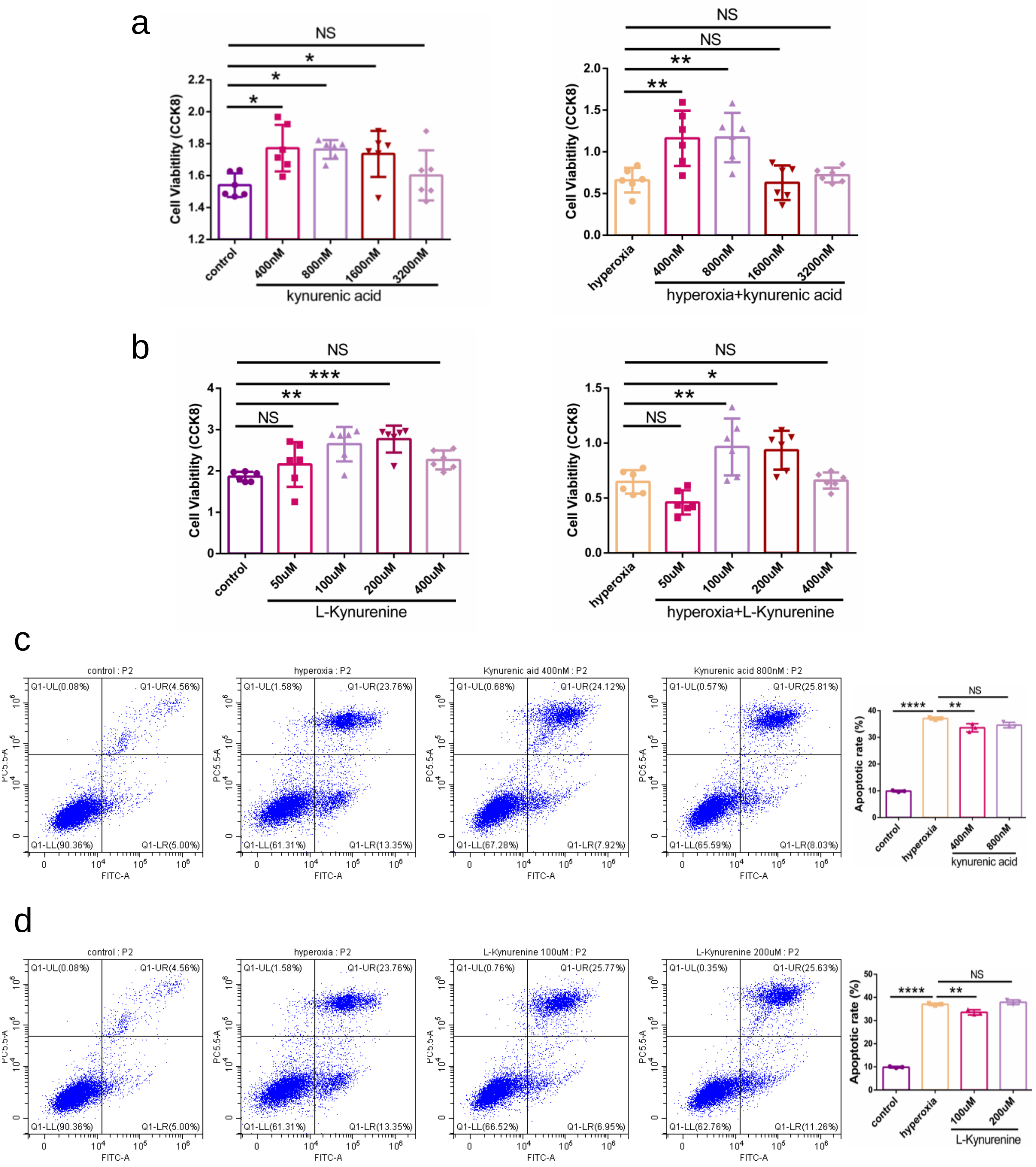

Supplementary Fig. 2. Protective effects of kynurenic acid and L-kynurenine on mouse lung epithelial (MLE-12) cells exposed to hyperoxia. (**a, c**) MLE-12 cells were treated with different doses of kynurenic acid (400, 800, 1600, 3200 nM) and L-kynurenine (50, 100, 200, 400 μM) for 48 h, respectively. Cell viability was detected by CCK-8 assay (n=6 each group). (**b, d**) MLE-12 cells were pretreated with different doses of kynurenic acid (400, 800, 1600, 3200 nM) and L-kynurenine (50, 100, 200, 400 μM), and then exposed to hyperoxia (80%-85%) for 48 h. Cell viability was detected by CCK-8 assay (n=6 each group). (**e-f**) Representative images of FACS analysis for cell apoptosis. MLE-12 cells were pretreated with different doses of kynurenic acid (400, 800 nM) and L-kynurenine (100, 200 μM) , and then exposed to hyperoxia (80%-85%) for 48 h. Quantification of the percentage of apoptotic cells (n=3 each group). Data are expressed as the means ± SD (**a-d**), and unpaired t-test was performed. Data are expressed as the means ± SD (**e-f**), and one-way ANOVA was performed. *** P<0.001; **P<0.01, * P<0.05, and NS: not statistically significant.

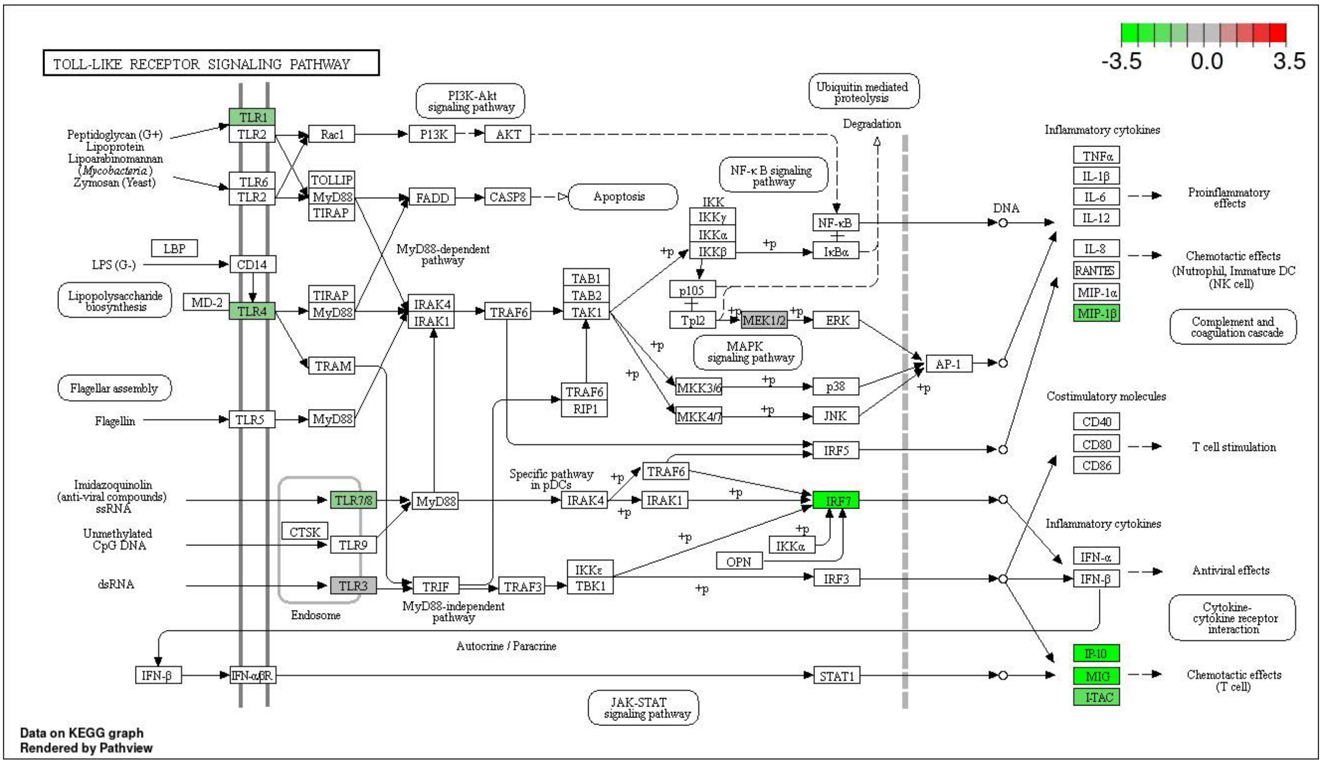

Supplementary Fig. 3. Genes changes of the lung RNA sequencing in Toll-like receptor signal pathway involved in present study.

Original blots presented in the manuscript

Fig 4i:

TLR4

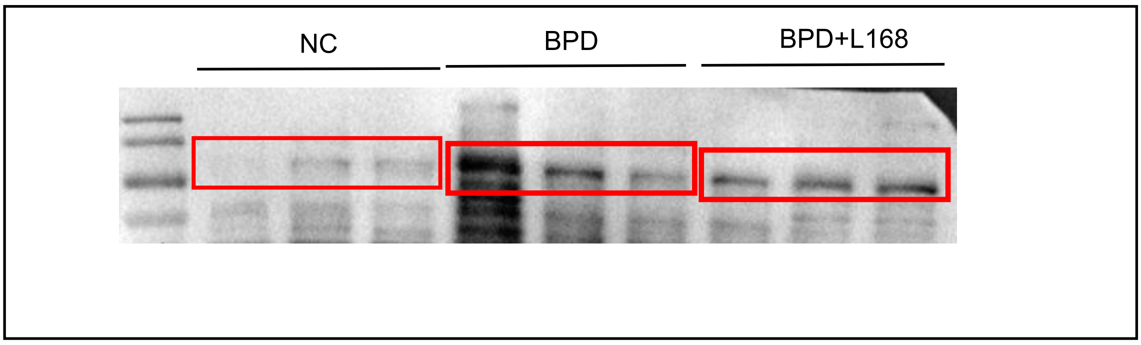

β-actin

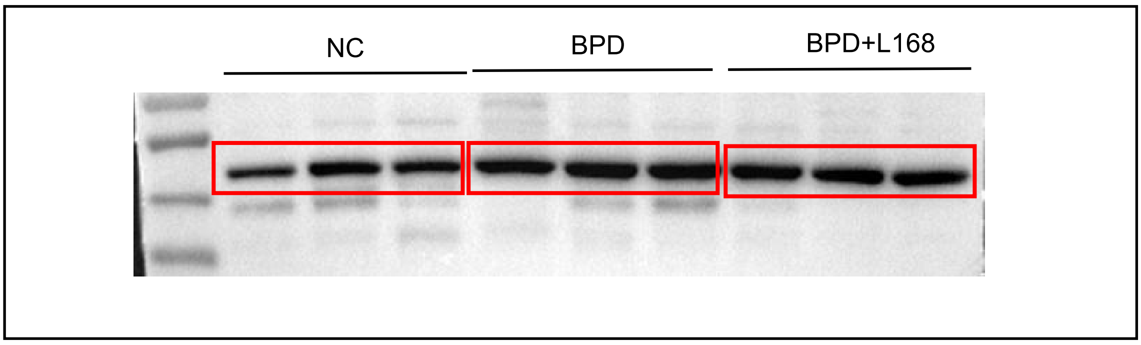

Fig 7b:

TLR4

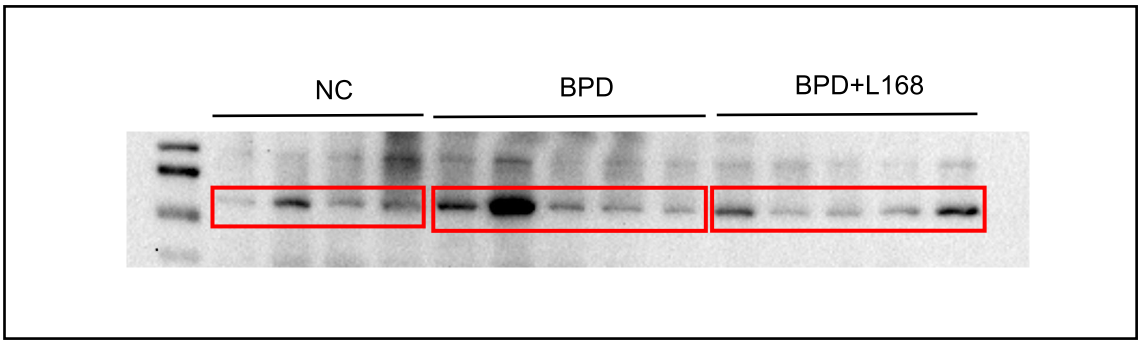

β-actin

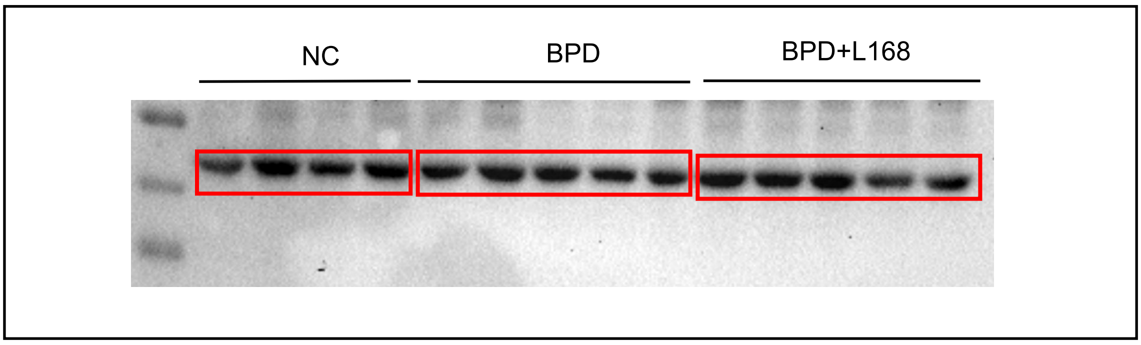

Fig 7c:

P65

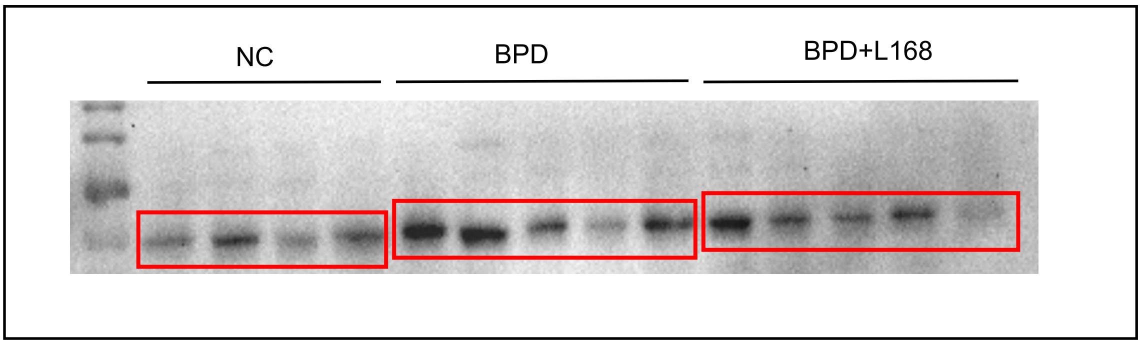

β-actin

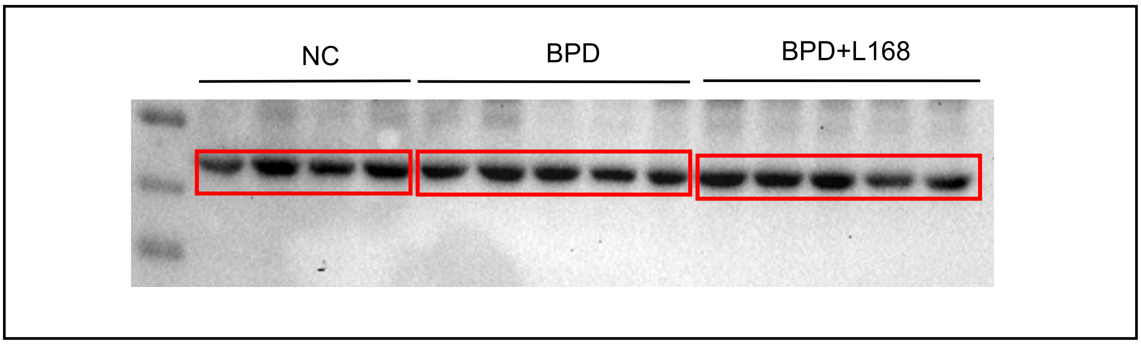

Fig 7d:

CCL4

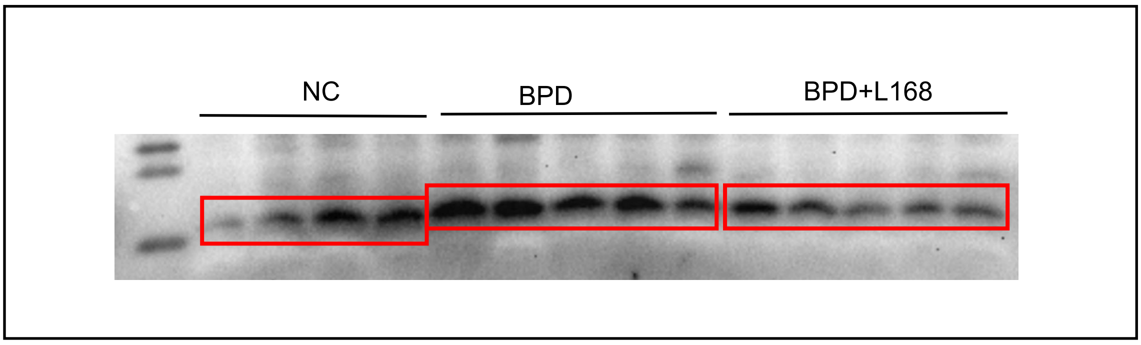

β-actin

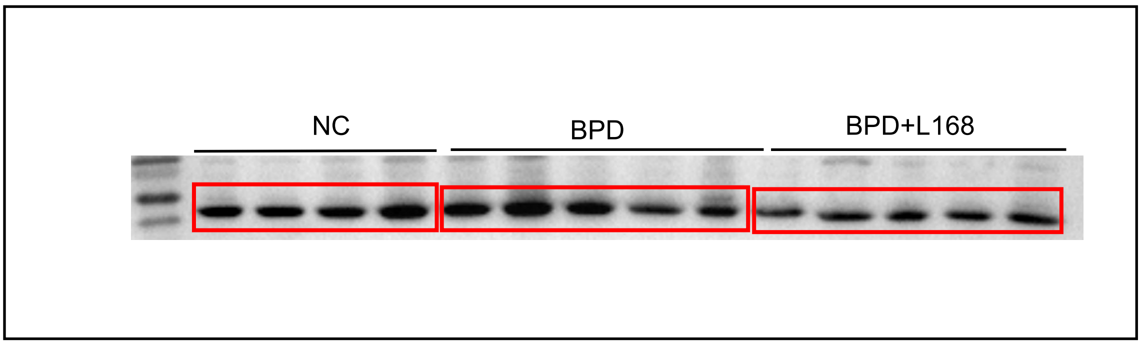

Supplement: Supplementary file 1 — Supplementary Figures [file 41522_2024_504_MOESM1_ESM.pdf]
